# Chemometric analysis of monoterpenes and sesquiterpenes of conifers

**DOI:** 10.3389/fpls.2024.1392539

**Published:** 2024-09-04

**Authors:** Eszter Bakó, Andrea Böszörményi, Bettina Vargáné Szabó, Marie Anne Engh, Péter Hegyi, Attila Ványolós, Dezső Csupor

**Affiliations:** ^1^ Department of Pharmacognosy, Semmelweis University, Budapest, Hungary; ^2^ Centre for Translational Medicine, Semmelweis University, Budapest, Hungary; ^3^ Institute of Clinical Pharmacy, University of Szeged, Szeged, Hungary; ^4^ Institute of Pancreatic Diseases, Semmelweis University, Budapest, Hungary; ^5^ Institute for Translational Medicine, University of Pécs, Pécs, Hungary

**Keywords:** conifers, gas chromatography, essential oils, chemotaxonomy, Pinaceae, Cupressaceae, terpenes, terpene synthases

## Abstract

Volatile organic compounds (VOCs) and essential oils of conifers are widely used in the pharmaceutical industry. This work aimed to analyze the VOCs of 30 conifer species representing the Pinaceae and Cupressaceae families. Samples were collected from arboreta in Hungary, and their chemical composition was determined by gas chromatography (SPME-GC/MS); then, chemometric analyses were performed using multivariate methods to identify characteristic VOCs of conifers. Here, we present results for monoterpene and sesquiterpene profiles of the examined conifer samples. The most abundant compounds detected were α-pinene, bornyl acetate, limonene, β-pinene, β-caryophyllene, β-myrcene, δ-3-carene, and β-phellandrene. The results showed that the following volatiles were characteristic of the conifer groups: sabinene (RRT=6.0) for the cupressoid group (which includes the Cupressaceae species), longifolene (RRT=15.0) and β-pinene (RRT=6.1) were characteristic of the pinoid group (including *Picea*, *Pinus*, and *Pseudotsuga* species), and camphene (RRT=5.5) and bornyl acetate (RRT=12.6) were characteristic of the abietoid group (including *Abies*, *Cedrus*, and *Tsuga* species). Our results on VOCs in the Pinaceae and Cupressaceae families contribute to the elucidation of biodiversity patterns of conifer species and, in addition, may support the industrial application of terpenes.

## 1 Introduction

Conifers are distributed worldwide from the tropics to the Arctic regions. Many species are economically important because they provide essential oils (EOs) and pine extracts, which are used in cosmetics as fragrances, in aromatherapy, or as food and beverage ingredients. In addition, the pharmaceutical industry uses EOs, resin, and the bark of certain conifer species. Juniper oil has a herbal monograph in the European Union, products containing pine tar have been used to treat different skin conditions (e.g., eczema, psoriasis, and skin inflammation), pine bark extracts have been investigated in a variety of chronic diseases, and the effects of pine resin have shown promise in the treatment of malignant ulcerating wounds (Barnes and Greive, 2017; Robertson et al., 2020; Batista et al., 2022).

EOs are mainly plant metabolites of terpenoids, i.e., mixtures of volatile organic compounds (VOCs). The building block of terpenoids is the isoprene unit, which comprises an isopentenyl skeleton. Monoterpenes, the most important essential oil components, consist of two isoprene units, while sesquiterpenes comprise three isoprene units. EOs and VOCs are important candidates for drug development. For example, monoterpenes are subjected to structural modifications in medicinal chemistry, a promising way to synthesize novel biologically active substances. According to Zielinska et al., borneol-based polymers have recently been investigated to improve cell adhesion, borneol isomers improve surface bacterial resistance properties, and α-pinene derivatives have been synthesized as potential antitumor agents, among others (Zielinska-Blajet and Feder-Kubis, 2020). Nootkatone, which is a sesquiterpene and has been used as a safe and effective active substance in a commercial insect repellent, was first isolated from *Chamaecyparis nootkatensis* (D.Don) Spach (Dietrich et al., 2006). Other economically important sesquiterpenes (e.g., β-farnesene, santalol, β-caryophyllene) are also available on the market. They are used in the food and fragrance industries due to their pleasant flavor and smell (Jiang and Wang, 2023). Sundar and Parikh, authors of a recent publication, have summarized the available knowledge on encapsulation techniques of essential oils. This process contributes to increasing the shelf life and the bioavailability of essential oils. Therefore, it is a promising technique for the development of EO-containing drugs (Sundar and Parikh, 2023). VOCs can also serve as initial molecules for the synthesis of other substances in the chemical industry. Oxygenated terpenoids may play an important role in the synthesis of biofuels (e.g., 1,8-cineole as the potential bio-jet fuel precursor) (Xie et al., 2020). Current applications of non-wood pine products are discussed in a study by Neis et al.; for example, sustainable thermoplastic elastomers are made from terpene-derived monomers (Neis et al., 2019). The wood of Cupressaceae is durable; therefore, it is a suitable source of timber and construction materials, particularly species from the *Calocedrus*, *Chamaecyparis*, *Cupressus*, and *Thuja* genera. Additionally, within the Cupressaceae family, *Chamaecyparis*, *Juniperus*, *Platycladus* and *Thuja* species are valued as ornamental trees. *Cryptomeria japonica* is the major plantation tree in the Far East. Furthermore, conifers are also used in horticulture, contributing significantly to their economic importance as cultivated species (Farjon, 2018; Mao et al., 2019).

The aim of this work was to investigate the monoterpene and sesquiterpene profiles of conifer species and to explore possible chemotaxonomic relationships. According to current gymnosperm phylogeny (Yang et al., 2022), extant gymnosperms are classified into three Linnaean classes, Cycadopsida, Ginkgoopsida, and Pinopsida - the latter including three subclasses, Gnetidae, Pinidae, and the Cupressidae. Conventionally, conifers are the last two subclasses. The classification of Gymnosperm (including subclasses and families) is presented in **Table 1**. Our current study focuses on two families of conifers, namely Pinaceae and Cupressaceae. The classification of these two families (i.e., a list of corresponding genera) and their habitats are presented in the **Supplementary Material** in **Supplementary Table S1** (WFO, 2024). Pinaceae is the most prominent gymnosperm family, with 11 genera and 272 species. It is widely distributed and restricted to the Northern Hemisphere (except for *P. merkusii*). The family is traditionally subdivided into two groups: the pinoid subfamily (Pinoideae) includes *Cathaya*, *Larix*, *Picea*, *Pinus*, and *Pseudotsuga*, whereas the abietoid subfamily (Abietoideae) includes *Abies*, *Cedrus*, *Keteleeria*, *Nothotsuga*, *Pseudolarix*, and *Tsuga* (Ran et al., 2018). Cupressaceae is also rich, comprising 31 genera and 169 species. It is distributed on all continents (except for Antarctica) from the sea level to the timberline. Gadek et al. identified seven subfamilies of the family: Cunninghamioideae, Taiwanioideae, Athrotaxidoideae, Sequoioideae, Taxodioideae, Callitroideae, and Cupressoideae. This classification was supported also by genome-based phylogeny studies (Gadek et al., 2000; Yang et al., 2012; Liu et al., 2022; Yang et al., 2022). Several samples investigated in the present study originate from species of the Cupressoideae subfamily, the largest subfamily within the Cupressaceae family, encompassing approximately 100 species distributed among 13 genera.

**Table 1:** Gymnosperm classification (Yang et al., 2022).

Classes	Subclasses	Family	No. of genera	No. of species
Ginkgoopsida	Ginkgoidae	Ginkgoaceae	1	1
Cycadopsida	Cycadidae	Cycadaceae	1	126
		Zamiaceae	9	255
Pinopsida	Cupressidae	Cephalotaxaceae	1	10
		**Cupressaceae**	31	169
		Araucariaceae	3	40
		Sciadopityaceae	1	1
		Taxaceae	5	29
		Podocarpaceae	20	181
	Gnetidae	Ephedraceae	1	70
		Gnetaceae	1	46
		Welwitschiaceae	1	1
	Pinidae	**Pinaceae**	11	272
**Total**			**86**	**1201**

Families indicated in bold are the subject of this study.

Most conifers are evergreen, although exceptions exist within certain genera, such as *Larix* and *Pseudolarix*, which are deciduous. The leaves of the Pinaceae are needle-like, whereas scale leaves are predominantly found in the Cupressaceae family. The morphological properties of the bark are characteristic of the species. Seeds are enclosed within cones, originating from the female cone, while the male cone produces pollens (Debreczy et al., 2011). Throughout various parts of conifer trees, oleoresin is present within resin ducts, resin vesicles, resin blisters, resin glands, and resin cells. Oleoresin is produced due to physical damage to the plant. Its primary purpose is chemical and physical defense against herbivores and to prevent the damaged part of the plant. Recent investigations have highlighted the involvement of terpenes in chemo-ecological interactions, as they influence insect behavior and serve as signal molecules for bark beetles. Additionally, alterations in the terpene profile contribute to enhanced resistance against microbial pathogens and insect-induced damage (Celedon and Bohlmann, 2019; Whitehill et al., 2019; Ninkuu et al., 2021).

In conifers, typical monoterpenes are α-pinene, β-pinene, camphene, 3-carene, myrcene, α-terpineol, bornyl acetate, and typical sesquiterpenes are β-caryophyllene and germacrene D (Bhardwaj et al., 2020). However, the similarities and differences in the essential oil composition of conifers have not been exhaustively investigated by chemometric methods. In our work, essential oil samples of 30 species of the Pinaceae and Cupressaceae families were analyzed chemically and chemometrically. Mapping the chemotaxonomic relationships of conifers can contribute to a better understanding of taxonomic relationships and provide valuable data for industrial exploitation of VOCs. In addition, this study may support other research on the terpene synthase system of conifers. A better understanding of the conifer terpene synthase system may contribute to the identification of new pathways for microbial production of terpenes and the design of key enzymes involved in the biosynthesis process (Zhang et al., 2017; Zhang and Hong, 2020).

## 2 Materials and methods

### 2.1 Plant material

Samples were collected in the Jeli Arboretum (9841 Kám, Hungary) in October 2020 and June 2021 and in the Folly Arboretum (8257 Badacsonyörs, Hungary) in May 2021. The plant material was placed in labeled polyethylene bags. The following parts of the plants were collected: needles (needles and branch tips), resin, cones, and bark. The species investigated are listed in **Table 2**, and the plant parts collected are listed in **Supplementary Table S2** in the **Supplementary Material**. The following species from the Pinaceae family were collected (18 species in total): *Abies concolor, Abies firma, Abies grandis, Abies holophylla, Cedrus atlantica, Picea omorika, Picea sitchensis, Pinus aristata, Pinus cembra, Pinus coulteri, Pinus heldreichii, Pinus nigra, Pinus peuce, Pinus pinaster, Pinus strobus, Pseudotsuga menziesii, Tsuga canadensis*, and *Tsuga heterophylla.* The following species from the Cupressaceae family were collected (12 species in total): *Calocedrus decurrens, Chamaecyparis pisifera, Cryptomeria japonica, Cupressus macnabiana, Juniperus chinensis, Juniperus communis, Juniperus drupacea, Juniperus rigida, Juniperus sabina, Juniperus virginiana, Sequoia sempervirens*, and *Thuja koraiensis.* Samples were conserved immediately after collection and then analyzed in the laboratory within 1-2 days. Needles were cut into small pieces (3-4 mm in length). If necessary due to their size, resin, cones, and bark samples were cut into smaller pieces to fit inside the headspace vials. Then the plant material was placed into individual vials, sealed with caps, and loaded into the GC/MS autosampler tray. Each vial was automatically transferred from the sample tray to the heated extraction chamber by the SMPE.

**Table 2:** The collected Pinaceae and Cupressaceae species and the place and date of the collection.

Jeli Arboretum, Kám, Hungary (October 2020)	Folly Arboretum, Badacsonyörs, Hungary (May 2021)	Jeli Arboretum, Kám, Hungary (June 2021)
*Abies concolor* (Gordon & Glend.) Lindl. ex Hildebr.	*Abies concolor* (Gordon & Glend.) Lindl. ex Hildebr.	*Abies concolor* (Gordon & Glend.) Lindl. ex Hildebr.
*Abies firma* Siebold & Zucc.	*Abies firma* Siebold & Zucc.	*Abies firma* Siebold & Zucc.
*Abies grandis* (Douglas ex D.Don) Lindl.	*Calocedrus decurrens* (Torr.) Florin	*Abies grandis* (Douglas ex D.Don) Lindl.
*Abies holophylla* Maxim.	*Cedrus atlantica* (Endl.) G.Manetti ex Carrière	*Calocedrus decurrens* (Torr.) Florin
*Cedrus atlantica* (Endl.) G.Manetti ex Carrière	*Cupressus macnabiana* A.Murray bis	*Cedrus atlantica* (Endl.) G.Manetti ex Carrière
*Chamaecyparis pisifera* (Siebold & Zucc.) Endl.	*Juniperus chinensis* L.	*Cryptomeria japonica* (Thunb. ex L.f.) D.Don
*Calocedrus decurrens* (Torr.) Florin	*Juniperus drupacea* Labill.	*Juniperus chinensis* L.
*Cryptomeria japonica* (Thunb. ex L.f.) D.Don	*Juniperus rigida* Siebold & Zucc.	*Juniperus communis* L.
*Picea omorika* (Pančić) Purk.	*Pinus coulteri* D.Don	*Juniperus sabina* L.
*Picea sitchensis* (Bong.) Carrière	*Pinus heldreichii* Christ	*Juniperus virginiana* L.
*Pinus aristata* Engelm.	*Pinus nigra* J.F.Arnold	*Picea sitchensis* (Bong.) Carrière
*Pinus cembra* L.	*Pinus pinaster* Aiton	*Pinus aristata* Engelm.
*Pinus heldreichii* Christ	*Pinus strobus* L.	*Pinus cembra* L.
*Pinus peuce* Griseb.	*Pseudotsuga menziesii* (Mirb.) Franco	*Pinus heldreichii* Christ
*Pinus strobus* L.	*Sequoia sempervirens* (D.Don) Endl.	*Pinus peuce* Griseb.
*Pseudotsuga menziesii* (Mirb.) Franco	*Tsuga canadensis* Carrière	*Pinus strobus* L.
*Sequoia sempervirens* (D.Don) Endl.	*Tsuga heterophylla* Sarg.	*Pseudotsuga menziesii* (Mirb.) Franco
*Thuja koraiensis* Nakai		*Sequoia sempervirens* (D.Don) Endl.
*Tsuga heterophylla* Sarg.		*Thuja koraiensis* Nakai
*Tsuga canadensis* Carrière		*Tsuga canadensis* Carrière
		*Tsuga heterophylla* Sarg.

### 2.2 SPME-GC/MS measurement

The chemical composition of the samples was measured by static headspace solid-phase microextraction (sHS-SPME) using gas chromatography-mass spectrometry (GC/MS). Prior to the analysis, conifer samples were placed into 20-mL headspace vials sealed with a silicon/PTFE septum.

#### 2.2.1 SPME

SPME, which is a fast technique that requires a low sample amount and operates without solvents, is suitable for the extraction of monoterpenes and sesquiterpenes (Knudsen et al., 1993; Szmigielski et al., 2011). SPME was performed using an automatic CTC Combi PAL multipurpose sampler (CTC Analytics AG, Zwingen, Switzerland). After a 5 minute incubation period at 100°C, a 65 μM carboxene/polydimethylsiloxane/divinylbenzene fiber (CAR/PDMS/DVB, StableFlex, Supelco, Bellefonte, PA, USA) was immersed into the headspace vial by the autosampler. The volatile components on the surface of the fiber were absorbed; the extraction was performed at 100°C for 20 minutes. Then, the fiber was transferred to the injector port of the GC/MS and desorbed (250°C, 1 minute). The injections were made in splitless mode. Then, the fiber was cleaned and conditioned in pure nitrogen (in Fiber Bakeout Station, 250°C, 15 min).

#### 2.2.2 GC

Analyses were performed using an Agilent 6890N/5973N GC/MSD (Santa Clara, CA, USA) system with a 30 m × 250 μm × 0.25 μm SLB-5MS capillary column (Supelco, Sigma-Aldrich, Philadelphia, PA, USA). The GC oven temperature was set to increase from 60°C (3 minutes isothermal) to 250°C at a rate of 8°C/min (1 minute isothermal). High-purity helium (6.0) (Messer) was used as the carrier gas at a 1.0 mL/min (37 cm/s) flow rate in constant flow mode.

#### 2.2.3 MS

The mass selective detector (MSD) was equipped with a quadrupole mass analyzer and operated in electron ionization mode (41–500 atomic mass units (amu) at 3.2 scan/s, at 70 eV, full scan mode).

#### 2.2.4 Evaluation

GC/MS measurement data were evaluated using MSD ChemStation D.02.00.275 software (Agilent, Santa Clara, CA, USA). Identification of volatile components was performed by comparing the calculated Kovats indexes to those found in the literature (Adams, 2007), and the NIST (National Institute of Standards and Technology) Chemistry WebBook (Linstrom) was also taken into account for identification of spectra. Percentage evaluation was performed by area normalization. This article summarizes the findings and measurement results for monoterpenes and sesquiterpenes and their derivatives, as our GC/MS method is not suitable for the proper identification of diterpenes.

### 2.3 Statistical analysis

The statistical analysis was performed to investigate the correlation between the chemical profiles of conifers and other attributes (such as species and plant organs). Calculations were performed using the SYN-TAX 2000 package (Podani, 2001).

#### 2.3.1 Principal component analysis

PCA is a dimension reduction method that helps to visualize the data structure in a few dimensions as efficiently as possible. The method creates new artificial variables (components) while the original information in the data is preserved. The object positions and the correlations between variables and components are simultaneously displayed in a coordinate system (biplot) to improve the visualization of results (Podani, 1997).

#### 2.3.2 Canonical variates analysis

CVA is also a multivariate technique used to evaluate the separation of observations into two or more groups and to calculate the contribution of each variable to this grouping (Podani, 2000). We investigated whether the three taxonomic groups (pinoid, abietoid, cupressoid) or plant organs (needles, cones, resin, bark) were separated and, if so, which volatile components could best interpret the separation. For the analyses, based on the PCA results, we selected the variables (VOCs) with the highest correlations with the first and the second principal components (see **Supplementary Material**, **Supplementary Tables S3**; **S4**).

#### 2.3.3 Analysis of variance

The total detected VOCs were analyzed by species with ANOVA, followed by a comparison of means using Tukey’s Honestly Significant Difference (HSD) test. The threshold of significance was set at *p*=0.05.

## 3 Results

### 3.1 GC/MS measurement results

A total of 151 conifer samples from 30 species collected from arboreta in Hungary were investigated using the SPME-GC/MS method. VOCs were analyzed in needles, bark, resin, and cone samples. All data, i.e., the identified volatile components of all samples, are presented in **Supplementary Table S5** in the **Supplementary Material**. Our current study focuses on monoterpenes, sesquiterpenes, and related derivatives, so unknown components or diterpenes are not included. A total of 183 VOCs were identified, with an average of 18 per sample.

Considering the results from all the 151 samples, we detected the following volatiles in the largest amount: α-pinene, bornyl acetate, limonene, β-pinene, β-caryophyllene, β-myrcene, δ-3-carene, β-phellandrene, longifolene, and germacrene D. The SPME-GC/MS measurements performed unequivocally demonstrate that terpenes are abundant compounds in conifers. Of the total VOCs, the most abundant component was α-pinene, and the largest amount of about 61% was detected in the resin of *Cupressus macnabiana*. Representative chromatograms of samples with high amounts of the above-mentioned terpenes can be found in the **Supplementary Material** (**Supplementary Figures S1-S5**).

Major VOCs detected in our samples (α-pinene, bornyl acetate, limonene, β-pinene, β-caryophyllene, sabinene, β-myrcene, and δ-3-carene) are the subject of medicinal and pharmaceutical research. The chemical structure, the chemical classification, and biological activities of the most abundant components are presented in **Table 3**.

**Table 3:** Chemical structure, chemical classification and biological activity of the most abundant volatile organic components measured in the samples.

The most abundant volatile components (VOCs) in conifer samples measured	Chemical structure of VOC	Chemical classification of VOC	Biological activities	References
α-pinene	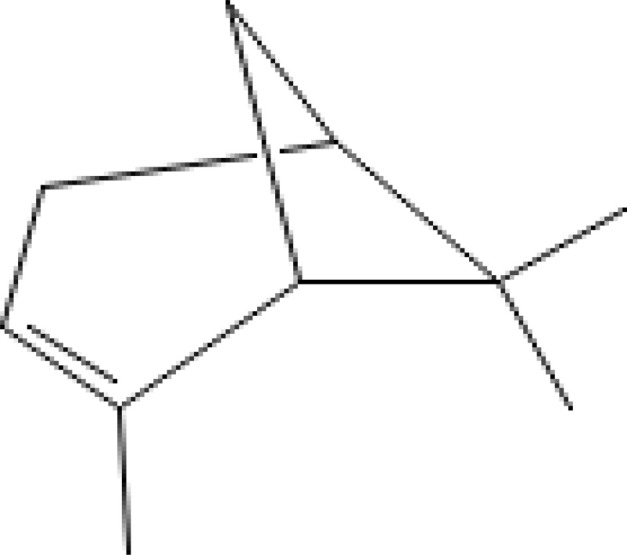	hydrocarbons, monoterpenes	antibacterial, antifungal, anti-inflammatory, antioxidative, neuroprotective, antitumor, etc.	(Allenspach and Steuer, 2021)
bornyl acetate	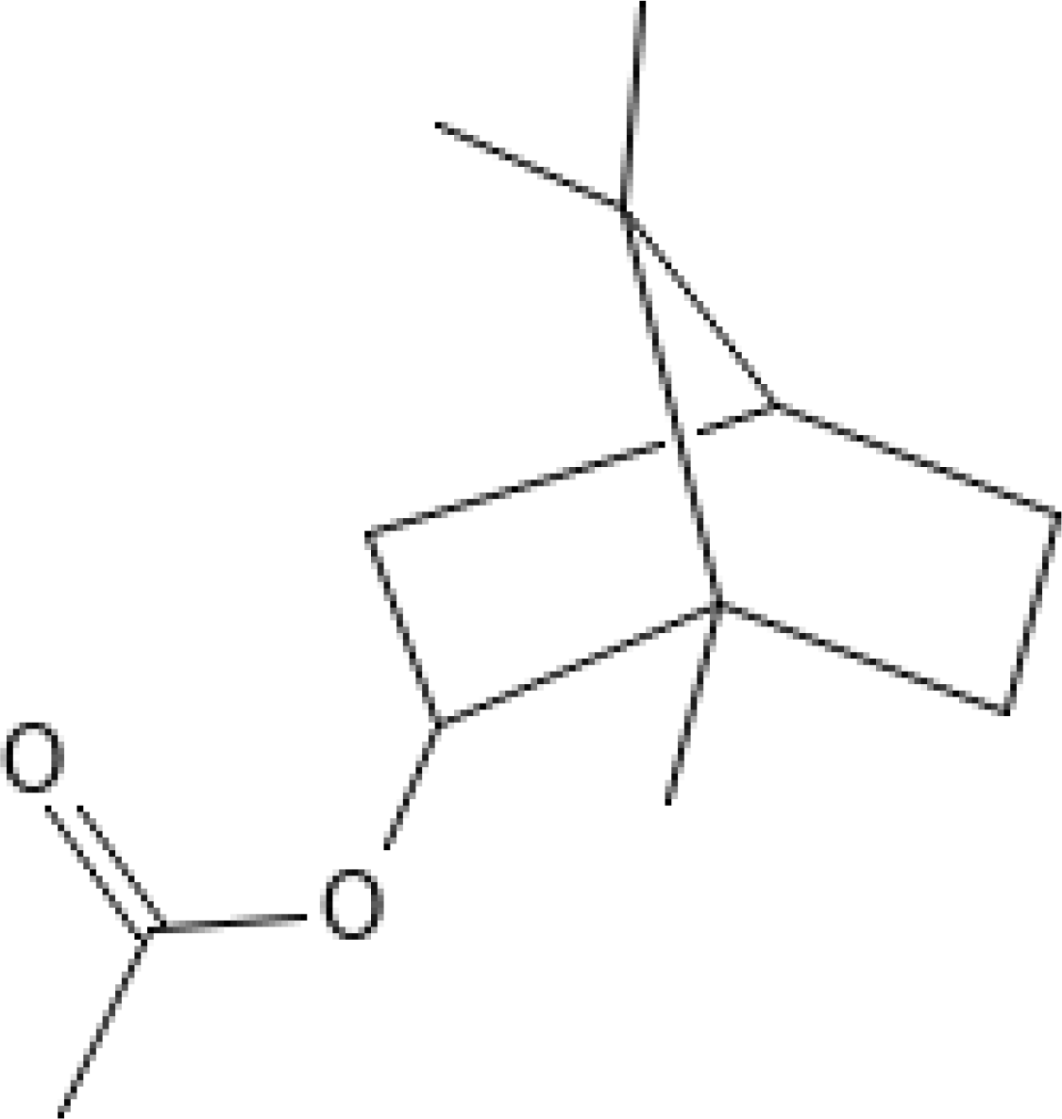	monoterpene esters	anti-inflammatory, immune-modulatory, sedative, etc.	(Zhao et al., 2023)
limonene	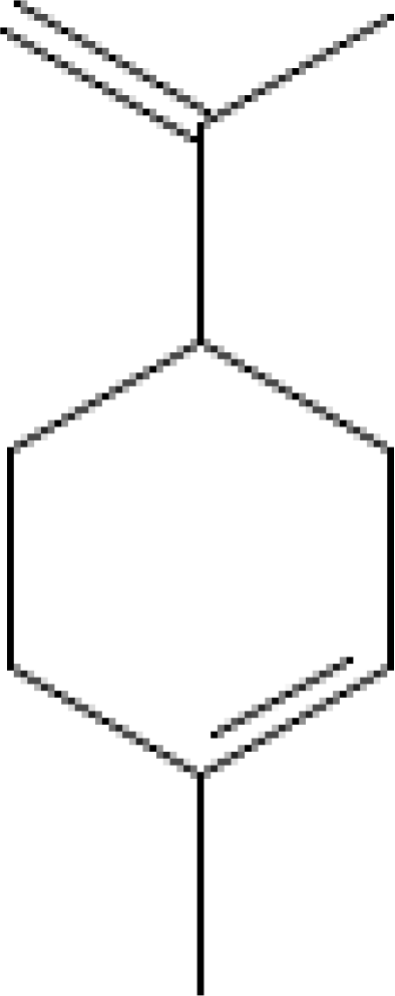	hydrocarbons, monoterpenes	antioxidant, antidiabetic, anticancer, anti-inflammatory, cardioprotective, immune-modulatory, etc.	(Anandakumar et al., 2021)
β-pinene	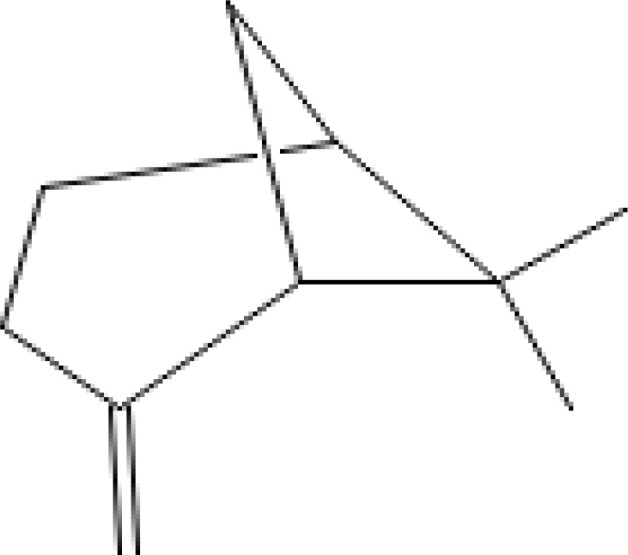	hydrocarbons, monoterpenes	antimicrobial, anticancer, anti-inflammatory, antiallergic, etc.	(Salehi et al., 2019)
β-caryophyllene	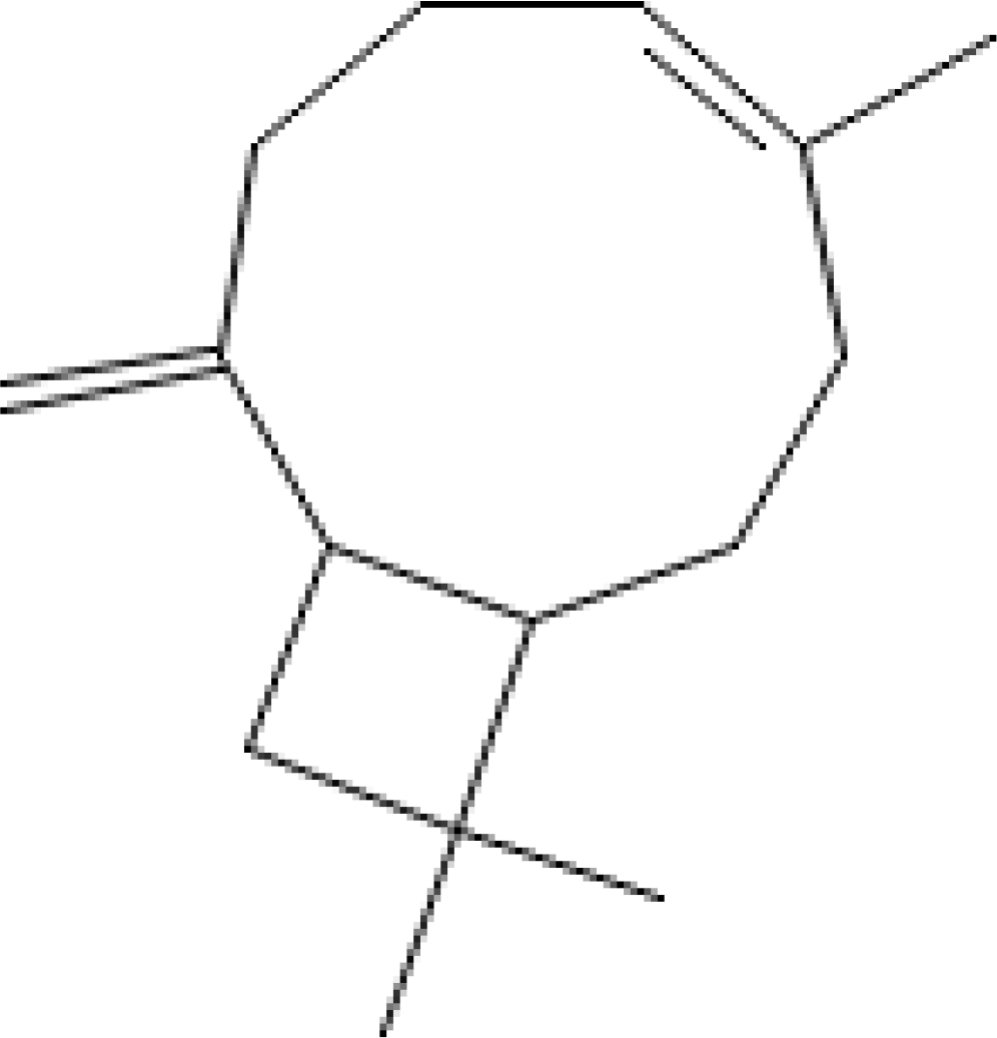	hydrocarbons, sesquiterpenes	antioxidant, anticancer, cardioprotective, immunomodulatory, antimicrobial activities, etc.	(Gyrdymova and Rubtsova, 2022)
sabinene	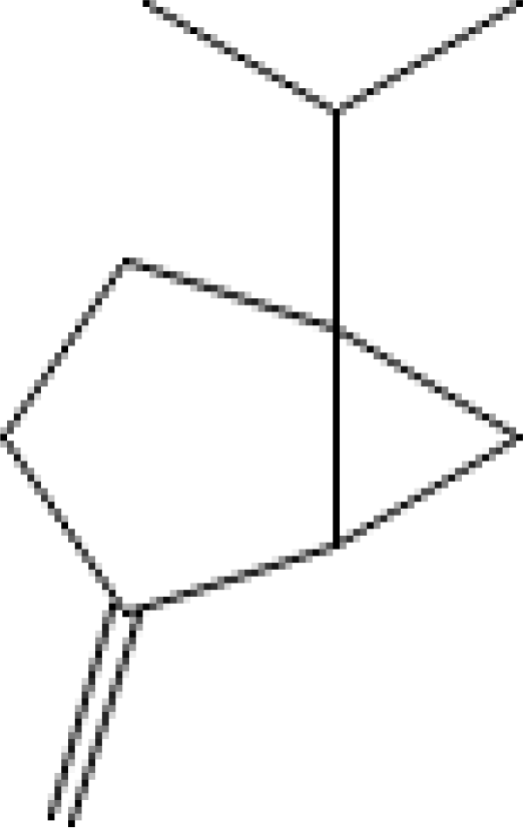	hydrocarbons, monoterpenes	anti-fungal, anti-inflammatory, etc.	(Cao et al., 2018)
β-myrcene	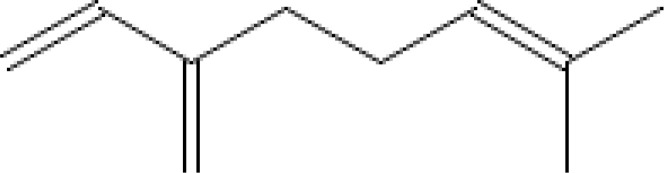	hydrocarbons, monoterpenes	anxiolytic, antioxidant, anti-aging, anti-inflammatory, analgesic, etc.	(Surendran et al., 2021)
δ-3-carene	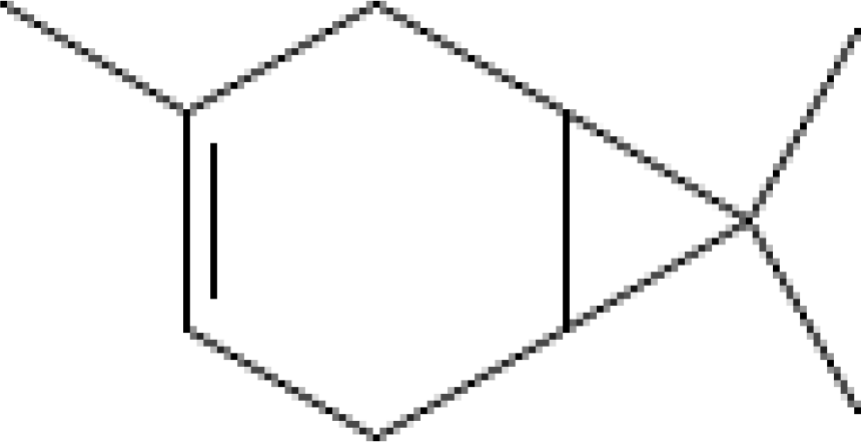	hydrocarbons, monoterpenes	anti-inflammatory	(Ocete et al., 1989)

The samples were collected from two arboreta (Folly Arboretum and Jeli Arboretum) in two consecutive years (2020 and 2021). We investigated the influence of the location and the collection time on the distribution of the VOCs in the collected samples. No difference was detected between the terpene profile of the samples from the two years (ANOVA, *p* = 1.00) and the two locations (ANOVA, *p* = 0.999).

Based on our GC/MS analysis on 103 samples from the Pinaceae family, the most abundant components were the following (mean value): α-pinene (15%), β-pinene (7%), bornyl acetate (7%), limonene (5%), β-caryophyllene (4%), β-phellandrene (4%), δ-3-carene (3%), longifolene (3%), β-myrcene (3%), and camphene (2%).

**Figure 1:**
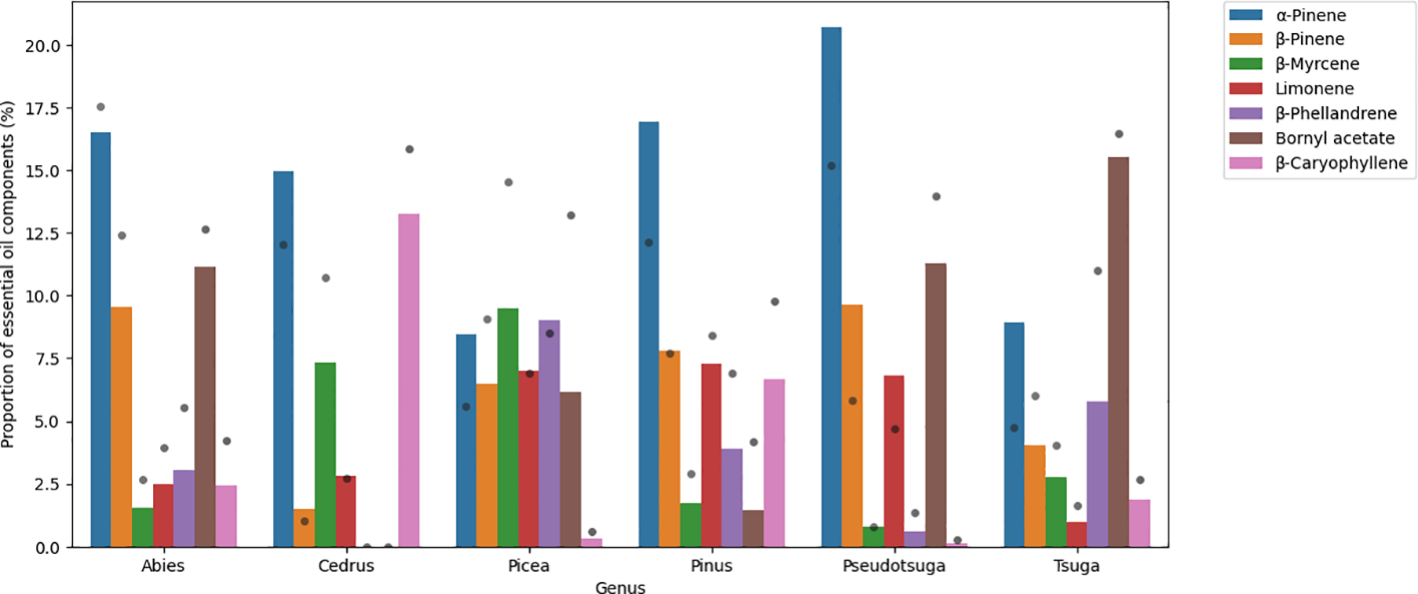
Bar plot showing the main volatile organic compounds of Pinaceae family. Results are mean area% measured by GC/MS. Grey dots represent standard deviation.

α-Pinene was abundant in each genus (8.4%-20.7%). β-Pinene can be found in large amounts in *Abies* (9.5%), in *Pinus* (7.8%) and in *Pseudotsuga* (9.7%) genera. β-Phellandrene concentration was high in *Picea* (9.0%) and in Tsuga (5.8%), and β-myrcene content was considerable in *Cedrus* (7.3%) and in *Picea* (9.5%) genera. Bornyl acetate levels were above 10% in the *Abies*, *Tsuga*, and *Pseudotsuga* genera (11.2%, 15.5%, and 11.3%, respectively). β-Caryophyllene was detected in large amounts in *Cedrus* (13.2%) and *Pinus* (6.7%), and limonene was measured to be around 7% in *Picea*, *Pinus*, and *Pseudotsuga* genera (7.0%, 7.3%, and 6.8%, respectively). For the respective bar plot, see **Figure 1**.

The most abundant components in the Cupressaceae family, based on the results of 48 samples, were (mean value): α-pinene (17%), limonene (7%), sabinene (5%), β-myrcene (4%), δ-3-carene (3%), bornyl acetate (3%), germacrene D (2%), terpinolene (2%), β-eudesmol (2%), and terpinene-4-ol (2%).

**Figure 2:**
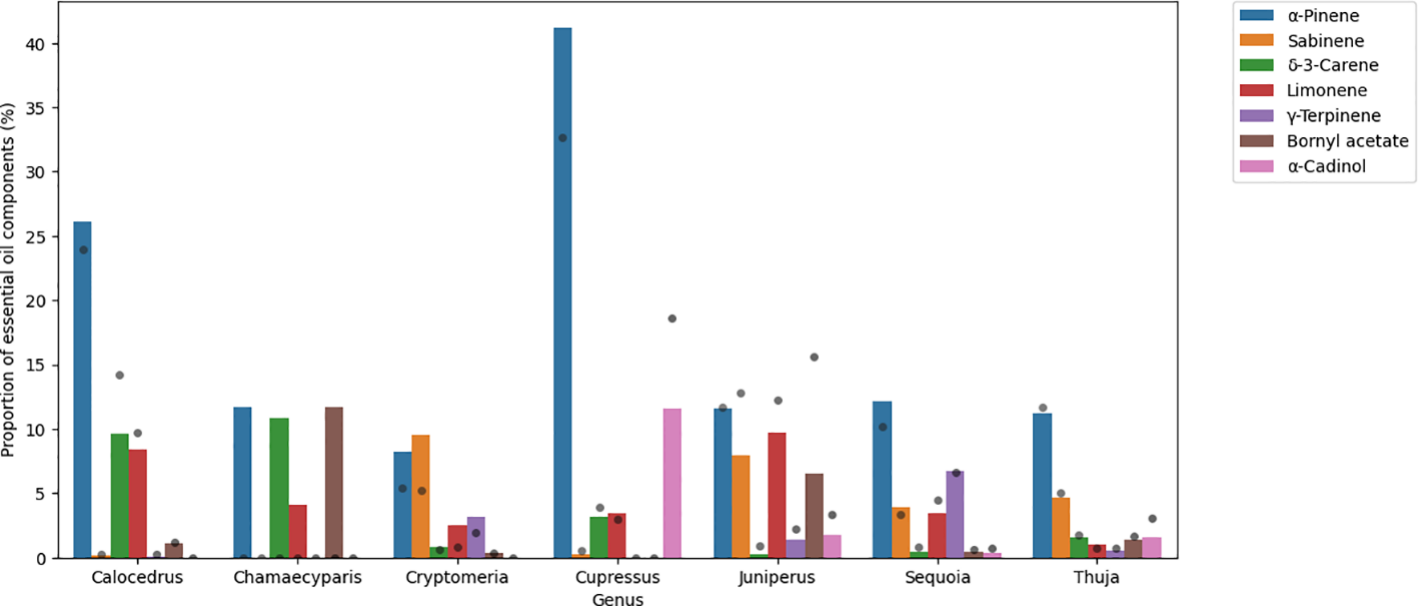
Bar plot showing the main volatiles of Cupressaceae family. Results are mean area% measured by GC/MS. Grey dots represent standard deviation.

*Cupressus* and *Calocedrus* genera contain high amounts of α-pinene (41.2% and 26.1%, respectively) and the other genera contain this VOC in large amounts as well (8.3%-12.2%). Sabinene was measured at around 10% in *Cryptomeria* and *Juniperus* genera (9.5% and 7.9%, respectively). δ-3-carene is abundant in *Calocedrus* (9.6%) and in *Chamaecyparis* (10.8%) genera. The amount of α-cadinol was high in the *Cupressus* genus (11.6%), and bornyl acetate was above 10% in the *Chamaecyparis* genus only (11.7%). For the bar plot, see **Figure 2**.

**Figure 3:**
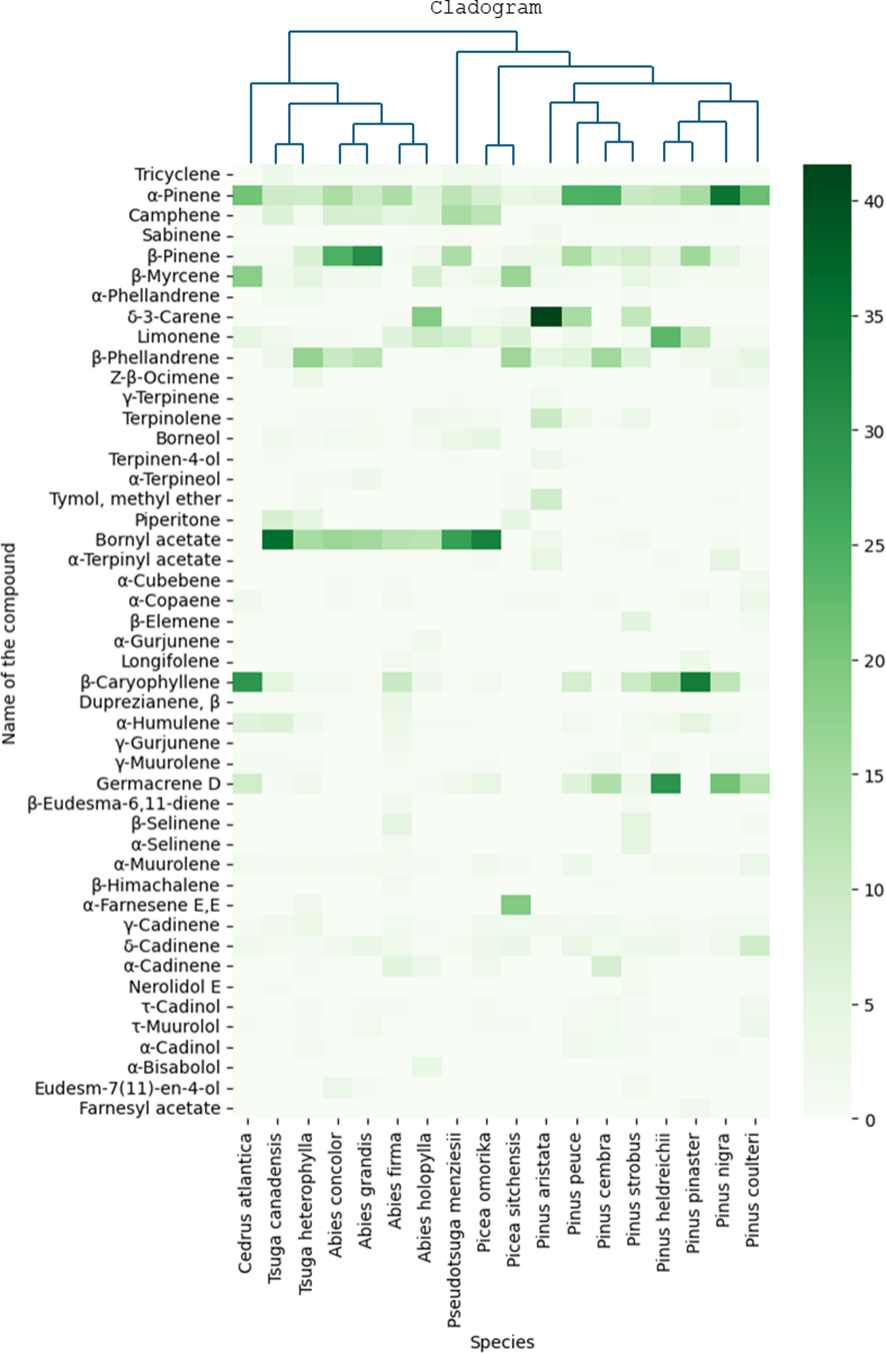
Heat map showing GC/MS results of all needle samples from the Pinaceae family together with the phylogeny of the species. Results are mean area% measured by GC/MS.

In the current study, 47 needle samples from species belonging to the Pinaceae family were collected and measured. To highlight the differences, we depicted the results on a heat map alongside the phylogeny of the species, see **Figure 3**. The cladogram presented in **Figure 3** was prepared based on the work of Stull et al. (2021). α-Pinene was detected in all samples, and its amount was considerable in all species (4%-35%). *P. nigra* (34.8%), *P. cembra* (24.9%), *P. peuce* (24.8%), *P. coulteri* (21.9%), and *C. atlantica* (20.8%) contained the highest percentage of α-pinene. Camphene was abundant in the species of *Picea omorika* (12%) and *Pseudotsuga menziesii* (15%). β-Pinene was also present in all species (1%-31%), with the highest percentage in *Abies grandis* and *Abies concolor* (30.8% and 24.7%, respectively). All species contained β-myrcene. *C. atlantica* and *P. sitchensis* contained the highest percentage (18% and 17%, respectively); however, the β-myrcene content remained below 8% in other species (0.5%-8%). Not all species contained δ-3-carene; only four species contained more than 10% of this VOC (*Abies holophylla* 19%, *P. aristata* 42%, *P. peuce* 15%, and *P. strobus* 11%). β-phellandrene was the major VOC of *P. sitchensis*, *P. cembra*, and *Tsuga heterophylla* (16%, 16%, and 17%, respectively). γ-Terpinene, terpinene-4-ol, terpinolene, and α-terpineol could not be detected in substantial amounts. Bornyl acetate was abundant in species of *Abies* (13%-16%), in *P. omorika* (33%), and in species of *Tsuga* and *Pseudotsuga* (15%-36%). Other species contained relatively low amounts of this VOC (0-3%). Almost all species contained β-caryophyllene; *C. atlantica* and *P. pinaster* had the highest levels of this VOC (30% and 34%, respectively). Germacrene D was measured to be above 10% in *P. nigra*, *P. heldreichii*, *P. cembra*, and *P. coulteri* (21%, 30%, 14%, and 13%, respectively), while other species contained lower amounts (0-9%). α-Muurolene, γ-cadinene, and δ-cadinene were not detected in high amounts (usually below 5%) but were present in most species.

### 3.2 Investigation of the relationships between volatiles and species by PCA

**Figure 4:**
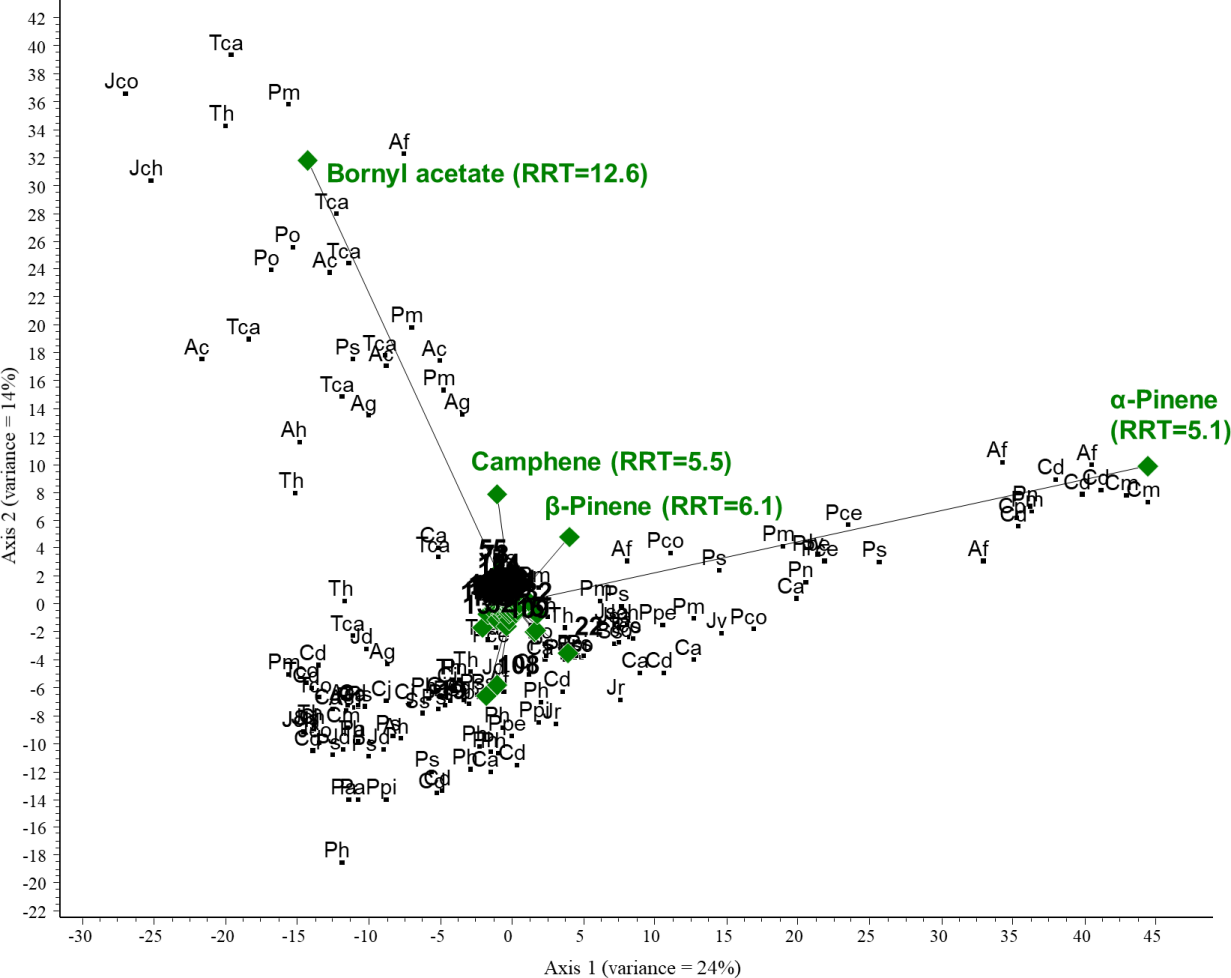
PCA biplot investigating the relationships between volatile organic compounds and plant species. Green diamonds are volatile components (RRT, relative retention time) and black dots are species. Ac, *Abies concolor*; Af, *Abies firma*; Ag, *Abies grandis*; Ah, *Abies holophylla*; Ca, *Cedrus atlantica*; Po, *Picea omorika*; Ps, *Picea sitchensis*; Pa, *Pinus aristata*; Pce, *Pinus cembra*; Pco, *Pinus coulteri*; Ph, *Pinus heldreichii*; Pn, *Pinus nigra*; Ppe, *Pinus peuce*; Ppi, *Pinus pinaster*; Ps, *Pinus strobus*; Pm, *Pseudotsuga menziesii*; Tca, *Tsuga canadensis*; Th, *Tsuga heterophylla*; Cd, *Calocedrus decurrens*; Cp, *Chamaecyparis pisifera*; Cj, *Cryptomeria japonica*; Cm, *Cupressus macnabiana*; JCh, *Juniperus chinensis*; Jco, *Juniperus communis*; Jd, *Juniperus drupacea*; Jr, *Juniperus rigida*; Jsa, *Juniperus sabina*; Jv, *Juniperus virginiana*; Ss, *Sequoia sempervirens*; and Tco, *Thuja koraiensis*.

**Figure 5:**
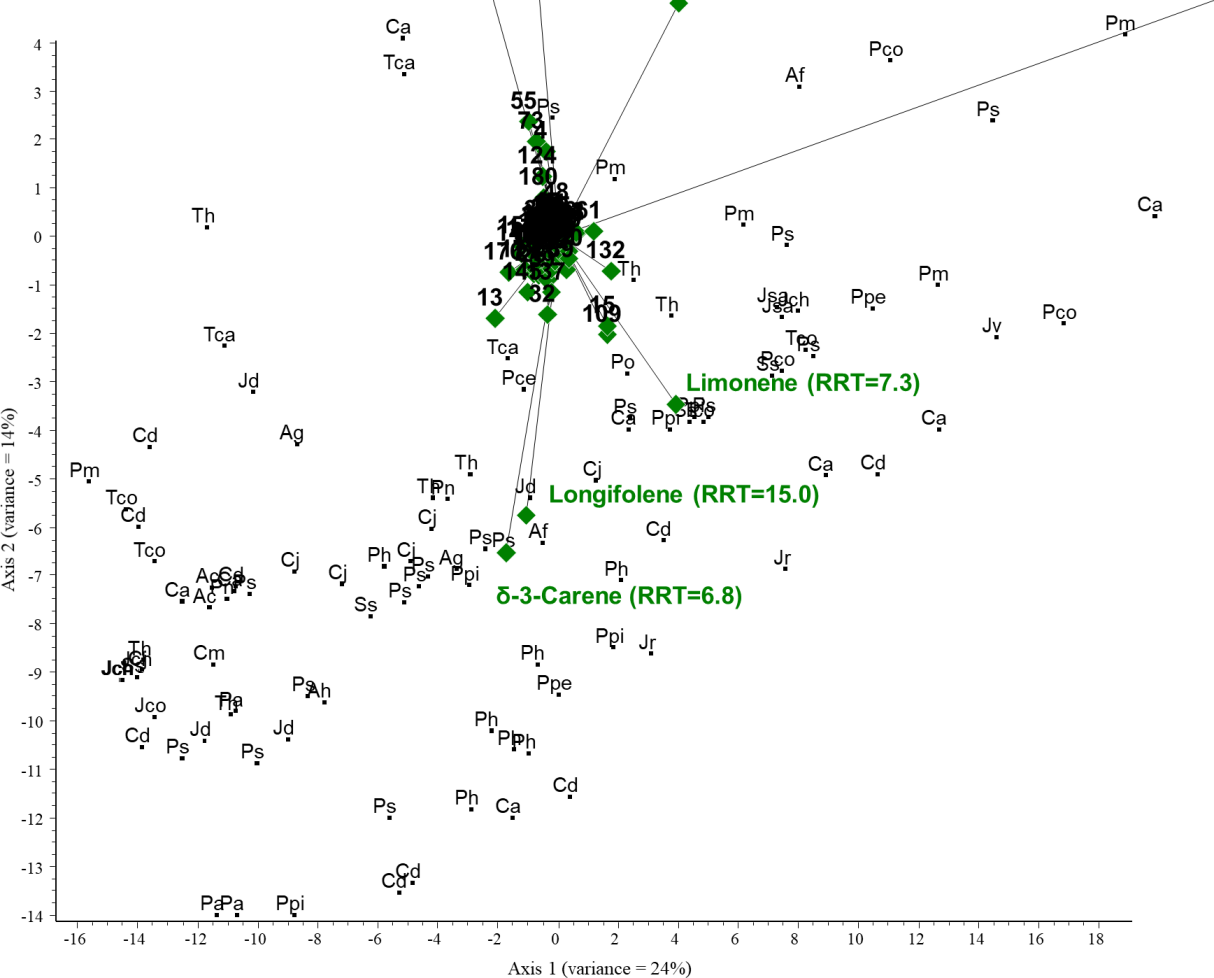
PCA biplot (scaled for visibility) investigating the relationships between volatile organic compounds and plant species. Green diamonds are volatile components (RRT, relative retention time) and black dots are species. Ac, *Abies concolor*; Af, *Abies firma*; Ag, *Abies grandis*; Ah, *Abies holophylla*; Ca, *Cedrus atlantica*; Po, *Picea omorika*; Ps, *Picea sitchensis*; Pa, *Pinus aristata*; Pce, *Pinus cembra*; Pco, *Pinus coulteri*; Ph, *Pinus heldreichii*; Pn, *Pinus nigra*; Ppe, *Pinus peuce*; Ppi, *Pinus pinaster*; Ps, *Pinus strobus*; Pm, *Pseudotsuga menziesii*; Tca, *Tsuga canadensis*; Th, *Tsuga heterophylla*; Cd, *Calocedrus decurrens*; Cp, *Chamaecyparis pisifera*; Cj, *Cryptomeria japonica*; Cm, *Cupressus macnabiana*; JCh, *Juniperus chinensis*; Jco, *Juniperus communis*; Jd, *Juniperus drupacea*; Jr, *Juniperus rigida*; Jsa, *Juniperus sabina*; Jv, *Juniperus virginiana*; Ss, *Sequoia sempervirens*; and Tco, *Thuja koraiensis*.

PCA was performed on the GC/MS results obtained for the conifer samples investigated. The PCA biplot revealed that two compounds were characteristic of the samples and thus represent the most considerable variance: α-pinene (RRT=5.1) and bornyl acetate (RRT=12.6). α-Pinene was the most correlated component with axis 1, and samples close to it contained the largest amounts of this compound. Bornyl acetate was highly correlated with axis 2; samples around it have the highest concentration. A large group of samples were accumulated around the origin; the above two compounds were largely missing in these samples. The total variance for the first two axes was 38%. See **Figures 4** and **5** (scaled for visibility).

**Figure 6:**
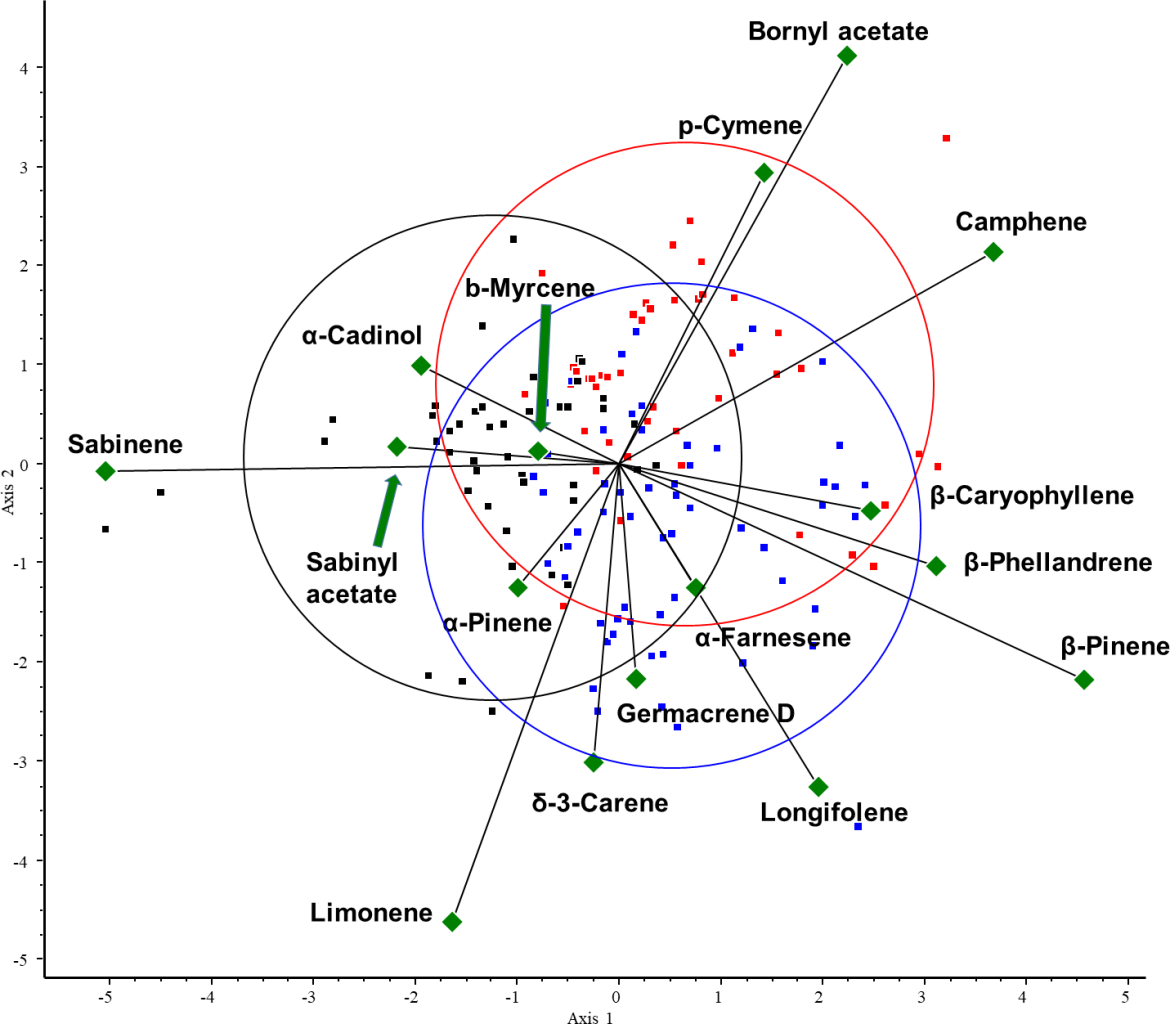
CVA biplot investigating the relationships between volatiles and species. CVA analysis was performed on all samples. Different groups are shown in different colors. Green triangles: volatile components. Red dots: abietoid group (containing *Abies*, *Cedrus*, and *Tsuga* species); blue dots: pinoid group (containing *Picea*, *Pinus*, and *Pseudotsuga* species); and black dots: cupressoid group (containing the Cupressaceae species).

Then, according to the PCA results, we selected the variables (VOCs) with the highest variance. To investigate the correlation between these VOCs and the three conifer groups, we performed CVA. The three groups were created based on the classification of the species: abietoid (containing *Abies*, *Cedrus*, and *Tsuga* species), pinoid (containing *Picea*, *Pinus* and *Pseudotsuga* species), and cupressoid groups (containing the Cupressaceae species). For the CVA plot, see **Figure 6**.

**Figure 7:**
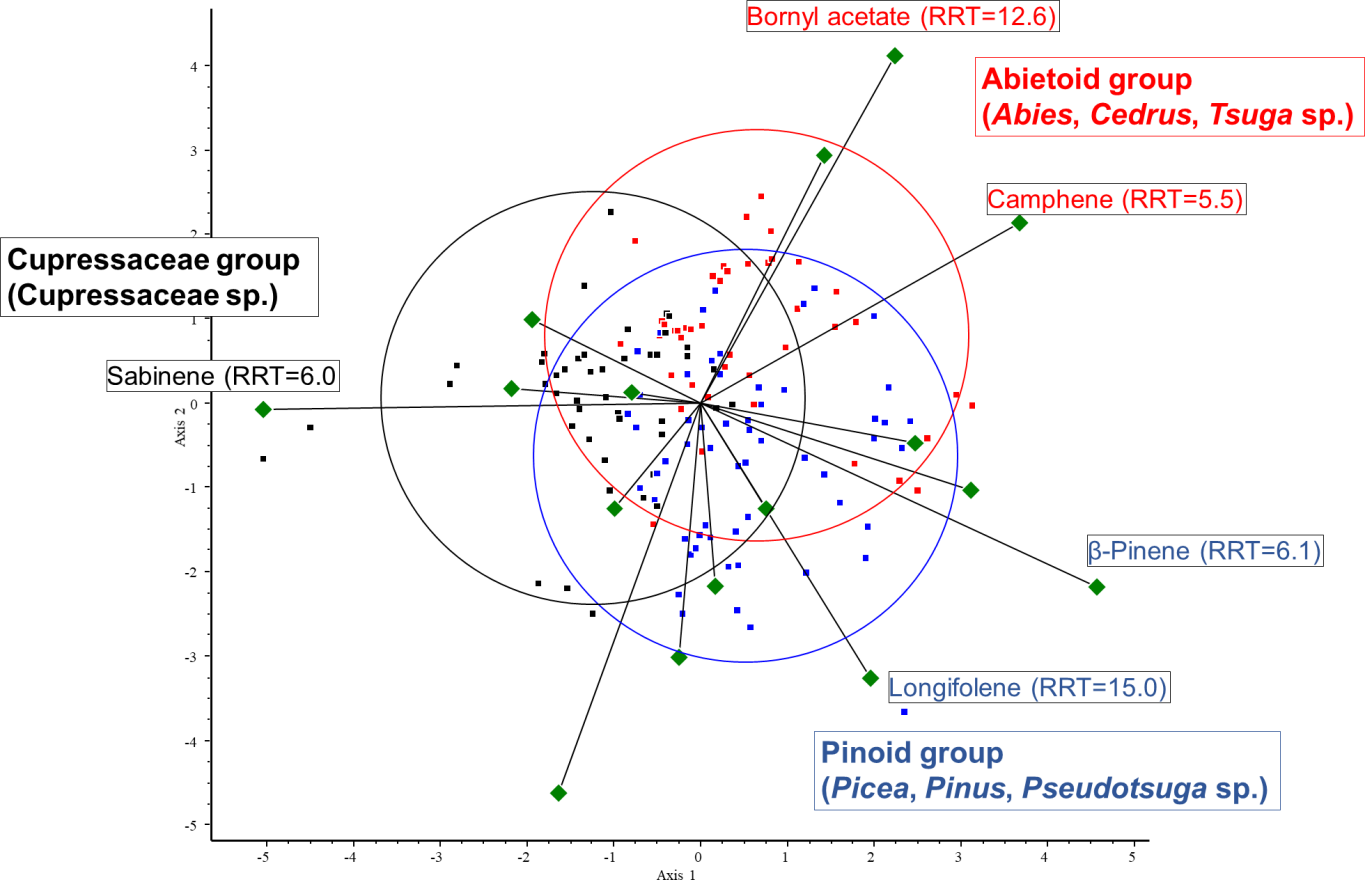
Main results of canonical variate analysis (CVA). Sabinene (RRT=6.0) is characteristic of the cupressoid group, longifolene (RRT=15.0) and β-pinene (RRT=6.1) are characteristic of the pinoid group, and camphene (RRT=5.5) and bornyl acetate (RRT=12.6) are characteristic of the abietoid group. RRT: relative retention time.

The results of the chemometric analyses indicate that the following volatiles are characteristic of certain groups: sabinene (RRT=6.0) is characteristic of the cupressoid group, longifolene (RRT=15.0) and β-pinene (RRT=6.1) are characteristic of the pinoid group, and camphene (RRT=5.5) and bornyl acetate (RRT=12.6) are characteristic of the abietoid group. The results show a negative correlation between the amount of bornyl acetate and limonene in the samples investigated (see **Figure 7**). Considering these 5 VOCs (sabinene, longifolene, β-pinene, camphene, and bornyl acetate), statistical analysis (Tukey HSD) showed a significant difference between the volatile profile of cupressoid and pinoid (*p*=0.023) and cupressoid and abietoid groups (*p*=0.0057). However, the difference was not statistically significant between the abietoid and cupressoid groups (*p*=0.776).

### 3.3 Investigation of the relationships between volatiles and collected plant organs of the Pinaceae family by PCA

**Figure 8:**
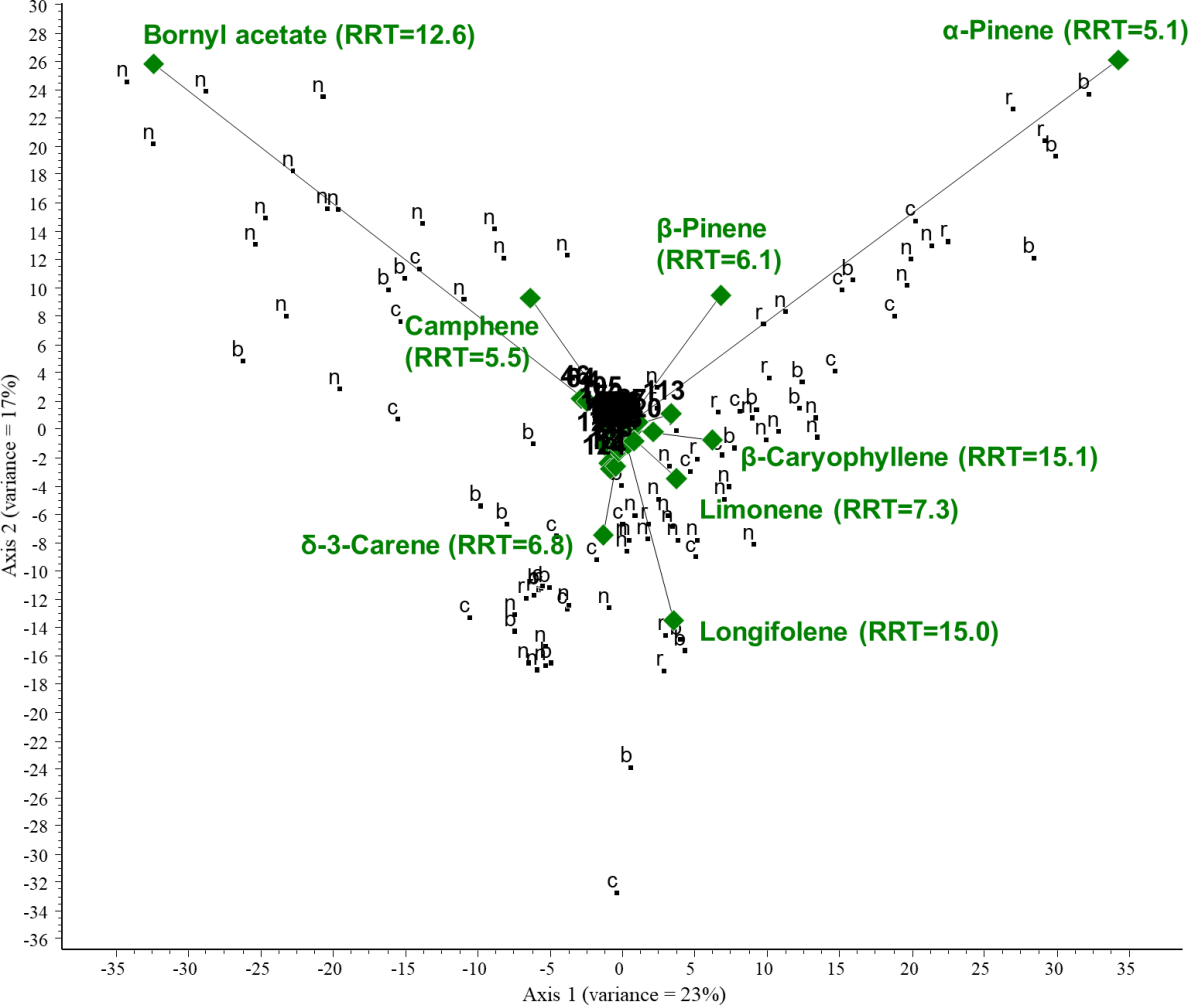
PCA biplot investigating the relationships between volatiles and collected plant organs of the Pinaceae family. Green diamonds are volatile components (RRT, relative retention time) and black dots are plant organs. n, needles; c, cones; r, resin; b, bark.

We performed centered PCA and then CVA to investigate the relationships between VOCs and collected plant organs of the Pinaceae family. The following plant organs were considered: needles, cones, bark, and resin. PCA was performed on all GC/MS data of the Pinaceae family. The first two ordination axes account for 40% of the total variance. Two compounds are responsible for a large portion of the variance in the data: α-pinene (RRT=5.1) and bornyl acetate (RRT=12.6). α-Pinene was present in large quantities in the samples. Samples near the arrowheads contained large amounts of the respective compound. Camphene and β-pinene are positively correlated, whereas longifolene is negatively correlated with axis 2. The results show a negative correlation between the amount of bornyl acetate and limonene in the samples investigated. For the PCA biplot, see **Figure 8**.

**Figure 9:**
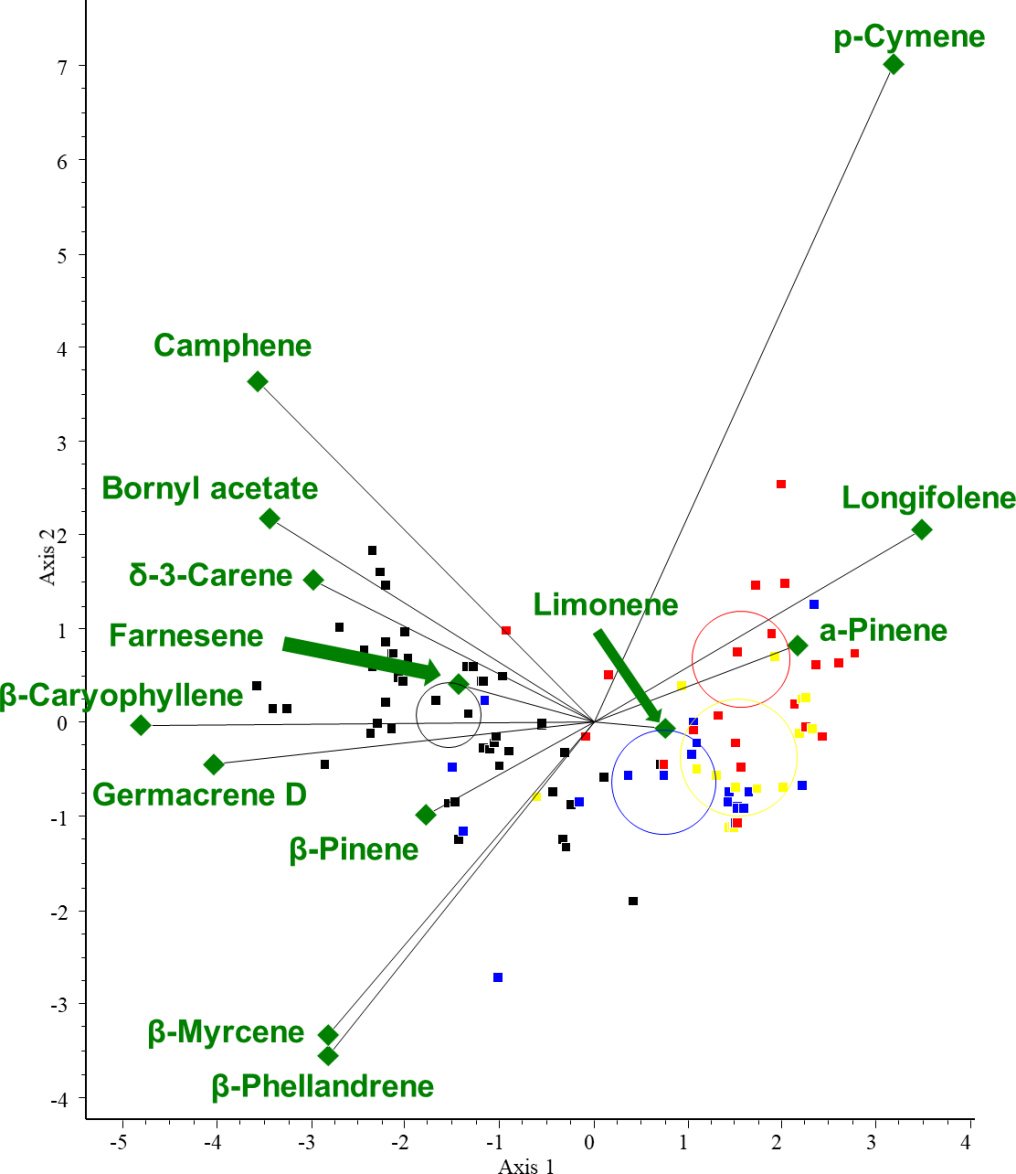
CVA biplot investigating the relationships between volatiles and collected plant organs of the Pinaceae family. Different groups are shown in different colors. Green diamonds: volatile components. Red dots: bark; blue dots: cones; black dots: needles; yellow dots: resin.

Then, to investigate the correlation between the VOCs with the highest variance and the plant organs, CVA was performed on the data. The results of the chemometric analyses show that β-caryophyllene (RRT=15.1) and germacrene D (RRT=16.2) are characteristic of needles, and their amounts are negatively correlated with α-pinene. Longifolene and α-pinene are characteristic of bark samples. There is also a negative correlation between the amounts of p-cymene and β-phellandrene or β-myrcene. For the CVA plot, see **Figure 9**. The chemical composition (mean area%) of samples of the Pinaceae family categorized by plant organs is presented in **Table 4** (only the highest values are shown).

**Table 4:** Volatile organic compounds expressed in the mean area% of samples measured by the SPME-GC/MS method in the Pinaceae family and categorized by plant organs.

Name of the compound (only the relevant components are listed)	*KI*	RRT	Chemical composition of samples investigated of the Pinaceae family (mean, area%)			
			resin	bark	cones	needles
			n=15	n=22	n=19	n=47
Tricyclene	914	4.9	0.1	0.2	0.1	0.7
α-Thujene	914	4.9	0.5	0.1	0.0	0.0
α-Pinene	923	5.1	19.8	17.2	13.1	12.6
Camphene	941	5.5	1.2	1.9	0.5	3.8
Sabinene	964	6.0	1.2	0.0	0.4	0.3
β-Pinene	968	6.1	6.2	5.1	6.6	8.3
β-Myrcene	977	6.3	1.0	0.3	4.4	4.5
δ-3-Carene	1003	6.8	0.6	1.7	1.3	5.6
α-Terpinene	1018	7.2	0.2	0.0	0.0	0.0
p-Cymene	1018	7.2	0.8	4.7	0.4	0.1
Limonene	1023	7.3	7.5	4.9	3.7	4.6
β-Phellandrene	1023	7.3	3.4	0.4	4.5	5.7
Terpinolene	1077	8.5	0.9	0.1	0.3	1.7
α-Campholenal	1119	9.4	0.3	0.1	0.7	0.0
trans-Pinocarveol	1133	9.7	0.7	0.6	1.3	0.0
cis-Verbenol	1138	9.8	0.8	0.3	1.1	0.0
Camphor	1143	9.9	0.0	0.7	0.0	0.0
Pinocarvone	1157	10.2	0.0	0.3	0.7	0.0
Borneol	1167	10.4	0.1	1.4	0.1	0.9
Terpinen-4-ol	1176	10.6	1.3	1.0	0.2	0.3
α-Terpineol	1190	10.9	1.7	2.6	0.2	0.3
Myrtenol	1190	10.9	0.6	0.2	0.7	0.0
Myrtenal	1190	10.9	0.0	0.2	1.3	0.0
Verbenone	1200	11.1	0.4	2.1	1.4	0.0
Thymol, methyl ether	1221	11.5	0.2	0.0	0.3	0.7
Piperitone	1247	12.0	0.0	0.0	0.0	0.8
Bornyl acetate	1279	12.6	1.4	5.0	4.1	10.6
α-Longipinene	1344	13.9	0.6	0.7	0.4	0.1
α-Copaene	1372	14.3	0.5	0.2	0.4	0.6
β-Bourbonene	1383	14.5	0.4	0.3	0.0	0.1
β-Elemene	1383	14.5	0.1	0.0	0.0	0.7
Longifolene	1412	15.0	3.6	7.7	4.8	0.4
β-Caryophyllene	1418	15.1	0.5	0.8	2.8	7.9
β-Farnesene	1441	15.5	0.6	0.1	0.5	0.1
α-Humulene	1453	15.7	0.1	0.1	0.9	2.1
γ-Muurolene	1476	16.1	0.6	0.6	0.5	0.6
Germacrene D	1482	16.2	0.1	0.1	1.6	4.3
β-Selinene	1492	16.4	1.0	0.4	0.2	0.9
α-Muurolene	1492	16.4	0.8	1.1	0.8	1.0
α-Farnesene	1492	16.4	0.0	0.0	0.0	1.4
α-Selinene	1500	16.5	0.0	0.0	0.0	0.6
β-Himachalene	1500	16.5	0.2	0.1	1.3	0.2
γ-Cadinene	1513	16.7	0.5	1.1	1.1	1.4
δ-Cadinene	1519	16.8	0.8	1.0	1.0	2.1
α-Cadinene	1538	17.1	0.0	0.3	0.0	1.1
Nerolidol	1550	17.3	0.8	0.2	0.0	0.2
Caryophyllene oxide	1588	17.9	0.2	0.5	1.0	0.1
τ-Cadinol	1640	18.7	0.0	0.7	0.1	0.5
τ-Muurolol	1653	18.9	0.0	0.6	0.2	0.6
α-Cadinol	1653	18.9	0.0	0.9	0.1	0.5
Eudesm-7(11)-en-4-ol	1667	19.1	0.1	1.1	0.1	0.4
Cadalene	1673	19.2	0.0	0.5	0.0	0.0

KI, calculated Kovats index; n, number of the measured samples; RRT, relative retention times.

## 4 Discussion

Terpenes represent a major group of plant metabolites in conifers. Monoterpenes and sesquiterpenes are essential oil components, whereas terpenoids with higher molecular weight, such as diterpenes, are usually not volatile. The volatile profiles of species are mainly determined by the terpene synthase (TPS) family (Alicandri et al., 2020); however, the actual amounts of volatiles in the plant part depend on different factors. These factors include developmental factors, stress, and other stimuli (temperature, biotic stress, pollution, season, etc.) (Kopaczyk et al., 2020).

We acknowledge that there are certain constraints on the results and conclusions because the sample tissues were collected from two artificially managed arboreta in the same region (Hungary). Whereas such a sampling scheme is not suitable for evaluating overall geographic variability in the volatile oil composition of any plant, it ensures that the trees were grown under very similar environmental conditions, thereby guaranteeing that our observations are less dependent on the environment. In this way, differences among species are more likely attributable to their own metabolism.

We reviewed the literature to compare our GC/MS results with the previously reported analyses. The major VOCs identified by our study in the abietoid group were α-pinene, β-pinene, β-caryophyllene, β-phellandrene and bornyl acetate, which is consistent with the literature. The major components found in our samples of *Abies concolor* (needles) were α-pinene, camphene, β-pinene, and bornyl acetate. In contrast to the analysis by Swor et al., the amount of δ-3-carene (0-1%) and limonene (0-2%) were negligible in our samples, but the other VOCs were found in similar amounts (Swor et al., 2022). In this analysis by Swor et al., the authors also provided the main chemical compounds of *A. grandis*, such as α-pinene, camphene, β-pinene, β-phellandrene, and bornyl acetate. They concluded that the investigated compositions were qualitatively similar to other *Abies* essential oils examined in the literature. The main components from our *A. grandis* samples were qualitatively similar. Sarria et al. identified an α/β pinene synthase in *A. grandis*, which produces about 40% α-pinene and about 60% of β-pinene (Sarria et al., 2014). This can also be a reasonable cause of the larger amount of β-pinene (31%) compared to α-pinene (10%) in our samples. According to Na-Hyun Lee at al., the major compounds of *Abies firma* were bornyl acetate, δ-3-carene, α-pinene, β-caryophyllene, and camphene (Lee et al., 2015). According to Satou at al. the main constituents of *A. firma* were α-pinene and δ-3-carene (Satou et al., 2011). The main VOCs of our *Abies firma* samples were consistent with the reported analyses, however a major difference was our samples containing only a low amount of δ-3-carene (0.7%). Based on the reported analysis of Lee and Hong, the main components in *A. holophylla* essential oil were bicyclo[2.2.1]heptan-2-ol (28%), δ-3-carene, α-pinene, camphene, limonene, β-myrcene (Lee and Hong, 2009). Bornyl acetate (bicyclo[2,2,1]-heptan-2-ol-1,7,7-trimethyl-acetate) was not detected by Lee and Hong, however our samples contained a considerable amount of this VOC (13%). Based on the above detailed chemical profile data of previous studies we can conclude that our results of *Abies* species align with those found in the literature. According to Boudarene et al., the main VOCs of *Cedrus atlantica* oils were α-pinene and β-caryophyllene. Our results are in line with these data, however we detected β-myrcene in considerable amount (18%). In contrast to other analyses reported in the literature, himachalenes were negligible in our samples (Boudarene et al., 2004; Ainane et al., 2019; Ailli et al., 2023). Bornyl acetate and α-pinene were reported as the most abundant VOCs of *Tsuga heterophylla* and *T. canadensis* species in the study of Lagalante (Lagalante and Montgomery, 2003). Correspondingly, our Tsuga *samples* contained a high percentage of α-pinene and bornyl acetate, however, the ratio of β-phellandrene was also high (17%) in our *T. heterophylla* samples. A study by Ankney et al. reported that one of the major components in *T. heterophylla* essential oil is β-phellandrene (6.6–19.3%) (Ankney et al., 2024). In summary, our findings are consistent with the literature.

The major VOCs in the pinoid group identified by us were α-pinene, β-pinene, bornyl acetate, β-phellandrene, β-caryophyllene and β-myrcene, which is consistent with the literature. Nikolic et al. reported that the main compounds of *Picea omorika* were bornyl acetate (29.2%), camphene (18.7%), and a-pinene (12.9%) (Nikolic et al., 2009). In our samples, the main components were the same, and their quantity closely matches the results of Nikolic et al. Furthermore, our results are consistent with the findings of Hall et al., as the main components of *P. sitchensis* were α-pinene, β-myrcene, limonene, β-phellandrene (Hall et al., 2011). However, in our samples, the amount of α-farnesene was high (19%). Farnesenes are reported to influence the behavior of insects, for example, *Picea sitchensis*, when attacked by pine weevils, releases α- and β-farnesenes (Kännaste et al., 2008). The main components of our *P. nigra* samples were α-pinene, germacrene D, and β-caryophyllene, which is in accordance with the results of Sarac et al. (2013); Koutsaviti et al. (2015), and Mitic et al. (2018), however Mitic et al. identified a relatively high percentage of β-pinene (10%) when compared to that of our samples (5%). Koutsaviti reported that their analyses were in line with the previous studies on the chemical composition of *P. nigra* needle oils. The most abundant VOCs of our *P. cembra* needle samples were α-pinene, β-pinene, and β-phellandrene, which is in line with the findings of Dormont et al. (1998). The major components of our samples in *P. heldreichii* were limonene, α-pinene, germacrene D, and β-caryophyllene. These data align with the results of Mitic et al. (2019) and Basholli-Salihu et al. (2017). *Pinus* aristata proved to contain the highest amount of δ-3-carene among our samples (42%), which is in good agreement with the findings described by Faiola et al. (2015). Despite the high variability, their analysis also suggests that the same seven compounds can be found in the largest amount in *Pinus aristata*, *Abies grandis*, and *Pseudotsuga menziesii*. The major components, as stated in the report, were: α-pinene, limonene, δ-3-carene, β-pinene, β-myrcene, camphene, and β-phellandrene. Our results do not confirm this statement, as *P. aristata* also contained terpinolene (10%) while *P. menziesii* and *A. grandis* contained bornyl acetate (28% and 16%, respectively). Major VOCs in our *P. peuce* samples were α-pinene, germacrene D, β-phellandrene and β-pinene and their quantities were similar as described in the literature (Nikolic et al., 2008; Basholli-Salihu et al., 2017; Mitic et al., 2018). However, δ-3-carene and β-caryophyllene were also considerable in our samples (15% and 8%, respectively). Corresponding to our results *P. strobus* contains high percentage of α-pinene, β-pinene, δ-3-carene, and β-caryophyllene. According to Koutsaviti et al. a considerable amount of β-pinene, α-pinene, and β-caryophyllene were identified in *P. strobus*. They concluded that quantitative differences were observed between their analysis and other analyses in the literature; however, qualitative similarities were observed (Krauze-Baranowska et al., 2002; Koutsaviti et al., 2015). The primary VOCs of our *P. menziesii* samples were bornyl acetate, camphene, and β-pinene. Based on the findings of Swor et al. (2023), the chemical profile of *P. menziesii* var. *glauca* is characterized by high levels of bornyl acetate and camphene, which is consistent with our results.

Sabinene is a typical component of the Cupressaceae family (Foster et al., 2013), and our results support this. In addition, α-thujone and β-thujone were detected in the Cupressaceae family but were absent in the samples of the Pinaceae family. Thujone is formed from sabinene, and sabinol is an intermediate product in thujone biosynthesis (Nemeth and Nguyen, 2020). In most cases where thujone was detected in our samples, sabinol was also present; see **Table 5** for all samples containing thujone.

**Table 5:** Presence of thujone and sabinol in relevant samples.

Sample (species, plant organ)	Volatile component (relative amount, %)			
	Sabinene (RRT=6.0)	α-Thujone (RRT=9.0)	β-Thujone (RRT=9.2)	Sabinol (RRT=9.7)
*Juniperus chinensis*, needles	5.1	0.0	1.0	0.7
*Juniperus sabina*, needles	16.4	0.0	1.3	2.9
*Juniperus chinensis*, needles	8.1	0.7	4.8	0.0
*Thuja koraiensis*, needles	8.7	1.0	7.9	0.4
*Thuja koraiensis*, needles	9.4	1.1	7.6	1.0

RRT, relative retention times.

Our research indicates differences in the terpene profiles of the plant organs of species from Pinaceae family (see **Figure 9**), which is in line with the literature (Dormont et al., 1998; Chizzola and Müllner, 2021; Butnaru et al., 2022). According to previous studies, the proportional amounts of several terpenes show strong positive correlations among different plant parts in conifers, or the same main VOCs could be identified in them (Dormont et al., 1998; Manninen et al., 2002). In addition, terpenoid production might be controlled not only by genetics, but also by environmental factors or plant organ development (de Simon et al., 2021; Bai, 2022). It is important to mention that although some research has observed significant differences in diterpenes (Chizzola and Müllner, 2021; de Simon et al., 2021; Nantongo et al., 2022), we did not investigate them in our study. The limitation of our study is that the collection of samples was not completely consistent (i.e., we did not always collect and analyze the same plant parts and did not consider the developmental stages of the plant organ). For future trials, we recommend using standardized and more comprehensive sample collections to improve the reliability of the data and reflect a more accurate composition of VOCs.

Terpenoid metabolites are synthesized in plants by specific terpene synthase enzymes. This is an enormous and intensively researched area (Karunanithi and Zerbe, 2019; Alicandri et al., 2020), and it is particularly important to investigate the relationships between the phylogeny and the terpene profiles of the species. This knowledge is necessary to refine metabolic engineering and synthetic biology tools to optimize the efficient production of economically valuable terpenoid compounds in the future. Our data on conifer VOCs can provide valuable scientific information for further research in this area.

## 5 Conclusion

In this comprehensive study, monoterpenes, sesquiterpenes, and their derivatives were analyzed by SPME-GC/MS from samples of 30 conifer species collected from arboreta in Hungary. A total of 151 samples (needles, cones, barks, and resin samples) were measured. Our gas chromatographic analyses in the Pinaceae family (103 samples) reveal that the most abundant components are α-pinene, β-pinene, bornyl acetate, limonene, and β-caryophyllene. In the Cupressaceae family (50 samples), the most important components are α-pinene, limonene, sabinene, β-myrcene, and δ-3-carene. In addition, further multivariate analyses (PCA and CVA) were performed on the analytical results to explore the correlation between chemical composition and species or plant organs. The detected VOCs showed similarities and differences within species and also among organs or higher taxonomic order levels such as genera. Characteristic chemical components were identified, such as sabinene for the cupressoid group, camphene and bornyl acetate for the abietoid group, and longifolene and β-pinene for the pinoid group.

Our present research performed on several conifer species provides comprehensive data on VOCs in the Pinaceae and Cupressaceae families. Furthermore, these data can complement our understanding of the biodiversity of conifer species and support other research on the production of terpenes by synthetic biology.

## Data Availability

The raw data supporting the conclusions of this article will be made available by the authors, without undue reservation.
