# Supplementary material for: Chemometric analysis of monoterpenes and sesquiterpenes of conifers
Source: Front Plant Sci. 2024 Sep 4;15:1392539. doi: 10.3389/fpls.2024.1392539 (PMC11408196; doi:10.3389/fpls.2024.1392539)
Supplement: Supplementary file 1 [file DataSheet1.docx]

Supplementary Material

Chemometric Analysis of Monoterpenes and Sesquiterpenes of Conifers

Eszter Bakó, Andrea Böszörményi, Bettina Szabó Vargáné, Marie Anne Engh, Péter Hegyi, Attila Ványolós^*^, Dezső Csupor^*^

*** Correspondence:**Dezső Csupor
csupor.dezso@szte.hu

Attila Ványolós
vanyolos.attila@semmelweis.hu

# Supplementary Figures and Tables

## Supplementary Tables

**Supplementary Table S1.** Classification of conifer families (WFO, 2024)

| **Family** | **Subfamily** | **Genus** | **Habitat** |
| --- | --- | --- | --- |
| Cupressaceae  (30 genera, 169 species) | Cunninghamioideae | *Cunninghamia* R.Br. | East and Southeast Asia |
|  | Taiwanioideae | *Taiwania* Hayata | Southeast Asia |
|  | Athrotaxidoideae | *Athrotaxis* D.Don | Tasmania |
|  | Sequoioideae | *Metasequoia* Hu & W.C.Cheng | Southeast Asia |
|  |  | *Sequoia* Endl. | Northwestern America |
|  |  | *Sequoiadendron* J.Buchholz | Northwestern America |
|  | Taxodioideae | *Cryptomeria* D.Don | Southeast Asia |
|  |  | *Glyptostrobus* Endl. | Southeast Asia |
|  |  | *Taxodium* Rich. | Northeast America, Mexico |
|  | Callitroideae | *Austrocedrus* Florin & Boutelje | South America (Argentina, Chile) |
|  |  | *Callitris* Vent. | Australia, New-Caledonia |
|  |  | *Diselma* Hook.f. | Tasmania |
|  |  | *Fitzroya* Hook.f. ex. Lindl. | South America |
|  |  | *Libocedrus* Endl. | New Zealand, New Guinea |
|  |  | *Papuacedrus* H.L.Li | New Guinea |
|  |  | *Pilgerodendron* Florin | South America (Argentina, Chile) |
|  |  | *Widdringtonia* Endl. | South Africa |
|  | Cupressoideae | *Callitropsis* Oerst. | Northwestern America |
|  |  | *Calocedrus* Kurz | Southeast Asia, Northwestern America |
|  |  | *Chamaecyparis* Spach | Southeast Asia, Northwestern America |
|  |  | *Cupressus* L. | Indo Mediterranean region |
|  |  | *Hesperocyparis* Bartel & R.A.Price | Northwestern America |
|  |  | Hesperotropsis Garland & Gerry Moore | Northwestern America |
|  |  | *Juniperus* L. | Northern Hemisphere |
|  |  | *Microbiota* Kom. | East Asia |
|  |  | *Tetraclinis* Mast. | Southwestern Europe, North Africa |
|  |  | *Thuja* L. | Southeast Asia, North America |
|  |  | *Thujopsis* Siebold & Zucc. ex. Endl. | East Asia, Japan |
|  |  | *Xanthocyparis* Farjon & T.H.Nguyên | Southeast Asia |
|  |  | *Platycladus* Spach | East and Southeast Asia |
| Pinaceae  (11 genera, 272 species) | Pinoideae | *Cathaya* Chun & Kuang | Southeast Asia (China) |
|  |  | *Larix* Mill. | Northern Hemisphere |
|  |  | *Picea* A.Dietr. | Northern Hemisphere |
|  |  | *Pinus* L. | Northern Hemisphere |
|  |  | *Pseudotsuga* Carrière | Southeast Asia, Taiwan, North America, Mexico |
|  | Abietoideae | *Abies* Mill. | Northern Hemisphere |
|  |  | *Cedrus* Trew | Indo Mediterranean region |
|  |  | *Keteleeria* Carrière | Southeast Asia (China, Taiwan, Vietnam, Laos) |
|  |  | *Nothotsuga* Hu ex. C.N.Page | Southeast Asia (China) |
|  |  | *Pseudolarix* Gordon | Southeast Asia (China) |
|  |  | *Tsuga* (Endl.) Carrière | Middle- and Southeast Asia, North America |

**Supplementary Table S2.** Collected plant organs of the species.

| **Jeli Arboretum, 2020** | | | | |
| --- | --- | --- | --- | --- |
| **Pinaceae family** | **needles** | **resin** | **cone** | **bark** |
| *Abies concolor* | x | - | - | x |
| *Abies firma* | x | - | - | x |
| *Pseudotsuga menziesii* | x | - | - | x |
| *Tsuga canadensis* | x | - | x | x |
| *Tsuga heterophylla* | x | - | x | x |
| *Pinus heldreichii* | x | - | x | x |
| *Pinus strobus* | x | - | - | x |
| *Cedrus atlantica* | x | x | x | - |
| *Abies holophylla* | x | - | - | - |
| *Picea omorika* | x | - | - | x |
| *Abies grandis* | x | - | - | x |
| *Pinus cembra* | x | - | - | - |
| *Pinus peuce* | x | - | - | x |
| *Pinus aristata* | x | - | - | - |
| *Picea sitchensis* | x | x | x | - |
| **Cupressaceae family** | **needles** | **resin** | **cone** | **bark** |
| *Cryptomeria japonica* | x | - | x | x |
| *Thuja koraiensis* | x | - | - | x |
| *Calocedrus decurrens* | x | - | x | x |
| *Sequoia sempervirens* | x | - | - | x |
| *Chamaecyparis pisifera* | x | - | - | x |
| **Folly Arboretum, 2021** | | | | |
| **Pinaceae family** | **needles** | **resin** | **cone** | **bark** |
| *Abies concolor* | x | x | x | x |
| *Abies firma* | x | - | - | - |
| *Pseudotsuga menziesii* | x | x | x | x |
| *Tsuga canadensis* | x | - | x | x |
| *Tsuga heterophylla* | x | x | x | x |
| *Pinus heldreichii* | x | x | x | x |
| *Pinus strobus* | x | x | - | x |
| *Cedrus atlantica* | x | - | x | x |
| *Pinus coulteri* | x | x | x | - |
| *Pinus nigra* | x | x | x | x |
| *Pinus pinaster* | x | - | x | x |
| **Cupressaceae family** | **needles** | **resin** | **cone** | **bark** |
| *Calocedrus decurrens* | x | - | x | x |
| *Sequoia sempervirens* | x | - | - | x |
| *Juniperus chinensis* | x | x | x | x |
| *Juniperus drupacea* | x | - | x | x |
| *Juniperus rigida* | x | - | - | - |
| *Cupressus macnabiana* | x | x | x | - |
| **Jeli Arboretum, 2021** | | | | |
| **Pinaceae family** | **needles** | **resin** | **cone** | **bark** |
| *Abies concolor* | x | x | - | x |
| *Abies firma* | x | x | - | x |
| *Pseudotsuga menziesii* | x | x | - | x |
| *Tsuga canadensis* | x | - | x | x |
| *Tsuga heterophylla* | x | x | - | x |
| *Pinus heldreichii* | x | x | - | - |
| *Pinus strobus* | x | x | - | x |
| *Cedrus atlantica* | - | x | x | - |
| *Abies grandis* | x | x | x | - |
| *Pinus cembra* | x | - | x | - |
| *Pinus peuce* | x | - | - | - |
| *Pinus aristata* | x | - | - | - |
| *Picea sitchensis* | x | x | x | x |
| **Cupressaceae family** | **needles** | **resin** | **cone** | **bark** |
| *Juniperus chinensis* | x | - | - | - |
| *Cryptomeria japonica* | x | - | - | x |
| *Thuja koraiensis* | x | x | - | - |
| *Calocedrus decurrens* | x | x | - | x |
| *Sequoia sempervirens* | x | - | - | x |
| *Juniperus communis* | x | - | x | - |
| *Juniperus sabina* | x | - | x | - |
| *Juniperus virginiana* | x | - | x | - |
| x: part of the plant was collected (it does not represent the number of sample collected);  -: the part of the plant was not collected | | | | |

**Supplementary Table S3.** Variables (volatile components) which showed the greatest variance (above 1%) by principal component analysis for the calculation of relationships between volatiles and species.

| **RRT** | **Volatile components** | **Variance**  **(AS%)** |
| --- | --- | --- |
| 5.1 | α-Pinene | 22 |
| 12.6 | Bornyl acetate | 13 |
| 6.8 | δ-3-Carene | 8 |
| 15.0 | Longifolene | 6 |
| 7.3 | Limonene | 6 |
| 6.1 | β-Pinene | 6 |
| 15.1 | β-Caryophyllene | 5 |
| 7.3 | β-Phellandrene | 4 |
| 6.3 | β-Myrcene | 4 |
| 6.0 | Sabinene | 3 |
| 16.2 | Germacrene D | 3 |
| 16.4 | α-Farnesene | 2 |
| 7.2 | p-Cymene | 2 |
| 5.5 | Camphene | 1 |
| 12.7 | Sabinyl acetate, trans | 1 |
| 18.9 | α-Cadinol | 1 |

**Supplementary Table S4.** Variables (volatile components) which showed the greatest variance (above 1%) by principal component analysis for the calculation of relationships between volatiles and collected plant organs of the Pinaceae family.

| **RRT** | **Volatile components** | **Variance**  **(AS%)** |
| --- | --- | --- |
| 5.1 | α-Pinene | 16 |
| 12.6 | Bornyl acetate | 15 |
| 15 | Longifolene | 9 |
| 6.8 | δ-3-Carene | 8 |
| 6.1 | β-Pinene | 7 |
| 15.1 | β-Caryophyllene | 7 |
| 7.3 | β-Phellandrene | 6 |
| 7.3 | Limonene | 5 |
| 6.3 | β-Myrcene | 5 |
| 16.2 | Germacrene D | 3 |
| 16.4 | α-Farnesene | 3 |
| 7.2 | p-Cymene | 2 |
| 5.5 | Camphene | 2 |

**Supplementary Table S5.**

*Supplementary Table S5 is uploaded separately.*

## Supplementary Figures

4.00

6.00

8.00

10.00

12.00

14.00

16.00

18.00

20.00

22.00

24.00

26.00

1000000

2000000

3000000

4000000

5000000

6000000

7000000

8000000

9000000

Time-->

Abundance

TIC: 4166 gyanta.D\data.ms

5.205

6.145

6.842

7.298

8.562

9.793

10.901

24.501

26.412

27.533

**Supplementary Figure S1.** Representative chromatogram of the resin of *Cupressus macnabiana* (measured by GC/MS). Most abundant volatile organic components: 61.3% of α-pinene (RRT=5.2), 7.7% of δ-3-carene (RRT=6.8), 4.2% of α-terpineol (RRT=10.9), and 3.1% of β-pinene (RRT=6.1).

4.00

6.00

8.00

10.00

12.00

14.00

16.00

18.00

20.00

22.00

24.00

26.00

2000000

4000000

6000000

8000000

1e+07

1.2e+07

1.4e+07

Time-->

Abundance

TIC: 9936 tu.D\data.ms

4.988

5.179

6.058

6.377

7.309

7.961

8.566

10.630

12.635

16.476

17.287

18.649

19.039

21.396

23.194

24.300

25.558

**Supplementary Figure S2.** Representative chromatogram of the needle of *Cryptomeria japonica* (measured by GC/MS). Most abundant volatile organic components: 11% of sabinene (RRT=6.0), 7.1% of α-pinene (RRT=5.1), 5.5% of terpinene-4-ol (RRT=10.6), and 4.3% of β-eudesmol (RRT=19.0). (The compound at RRT=24.3 is a diterpene molecule; diterpenes are not discussed by this article.)

4.00

6.00

8.00

10.00

12.00

14.00

16.00

18.00

20.00

22.00

24.00

26.00

1000000

2000000

3000000

4000000

5000000

6000000

7000000

8000000

Time-->

Abundance

TIC: 7117 tu.D\data.ms

4.168

5.174

6.385

6.892

7.313

8.579

11.372

12.851

13.781

15.171

15.455

16.733

18.353

19.017

**Supplementary Figure S3.** Representative chromatogram of the needle of *Calocedrus decurrens* (measured by GC/MS). Most abundant volatile organic components: 31.6% of δ-3-carene (RRT=6.8), 12.8% of α-pinene (RRT=5.1), 11.5% of β-myrcene (RRT=6.3), 11.4% of terpinolene (RRT=8.5), and 2.5% of β-caryophyllene (RRT=15.1).

4.00

6.00

8.00

10.00

12.00

14.00

16.00

18.00

20.00

22.00

24.00

26.00

0

1000000

2000000

3000000

4000000

5000000

6000000

7000000

8000000

9000000

Time-->

Abundance

TIC: 38 tu.D\data.ms

4.986

5.170

6.053

6.369

7.303

7.952

8.562

10.610

12.633

16.797

7.111

17.282

18.644

19.043

21.396

23.190

24.291

25.554

**Supplementary Figure S4.** Representative chromatogram of the needle of *Cryptomeria japonica* (measured by GC/MS). Most abundant volatile organic components: 11.7% of sabinene (RRT=6.0), 7% of β-eudesmol (RRT=19.0), 5.9% of α-pinene (RRT=5.1), 2.8% of limonene (RRT=7.3), 2.5% of δ-cadinene (RRT=16.8), and 2.3% of elemol (RRT=17.2). (The compound at RRT=24.3 is a diterpene molecule; diterpenes are not discussed by this article.)

4.00

6.00

8.00

10.00

12.00

14.00

16.00

18.00

20.00

22.00

24.00

26.00

0

1000000

2000000

3000000

4000000

5000000

6000000

7000000

8000000

Time-->

Abundance

TIC: 2608 tu.D\data.ms

4.931

5.162

5.531

6.154

7.301

7.336

10.445

12.657

13.817

16.478

16.798

18.523

19.184

**Supplementary Figure S5.** Representative chromatogram of the needle of *Abies concolor* (measured by GC/MS). Most abundant volatile organic components: 29.2% of bornyl acetate (RRT=12.6), 13.6% of β-pinene (RRT=6.1), 9.7% of camphene (RRT=5.5), 9.2% of α-pinene (RRT=5.1), and 5.3% of
δ-cadinene (RRT=16.8).
